# Specific IgG glycosylation differences precede relapse in PR3-ANCA associated vasculitis patients with and without ANCA rise

**DOI:** 10.3389/fimmu.2023.1214945

**Published:** 2023-09-29

**Authors:** Iwona Wojcik, Manfred Wuhrer, Peter Heeringa, Coen A. Stegeman, Abraham Rutgers, David Falck

**Affiliations:** ^1^ Center for Proteomics and Metabolomics, Leiden University Medical Center, Leiden, Netherlands; ^2^ Glycoscience Research Laboratory, Genos Ltd., Zagreb, Croatia; ^3^ Department of Pathology and Medical Biology, University Medical Center Groningen, Groningen, Netherlands; ^4^ Division of Nephrology, Department of Internal Medicine, University Medical Center Groningen, Groningen, Netherlands; ^5^ Department of Rheumatology and Clinical Immunology, University Medical Center Groningen, Groningen, Netherlands

**Keywords:** immunoglobulin G, fucosylation, glycopeptides, glycosylation, anti-neutrophil cytoplasmic antibody–associated vasculitis, mass spectrometry

## Abstract

**Introduction:**

Immunoglobulin G (IgG) contains a conserved N-glycan in the fragment crystallizable (Fc), modulating its structure and effector functions. In anti-neutrophil cytoplasmic antibody (ANCA)-associated vasculitis (AAV) alterations of IgG Fc-glycosylation have been observed to correlate with the disease course. Here, we examined longitudinal changes in N*-*linked Fc glycans of IgG in an AAV patient cohort and their relationship with disease flares.

**Methods:**

Using liquid chromatography coupled with mass spectrometry, we analysed IgG Fc-glycosylation in 410 longitudinal samples from 96 individuals with AAV.

**Results:**

Analysis of the cross-sectional differences as well as longitudinal changes demonstrated that IgGs of relapsing PR3-ANCA patients have higher ΔFc-bisection at diagnosis (*P =* 0.004) and exhibit a decrease in Fc-sialylation prior to the relapse (*P =* 0.0004), discriminating them from non-relapsing patients. Most importantly, PR3-ANCA patients who experienced an ANCA rise and relapsed shortly thereafter, exhibit lower IgG Fc-fucosylation levels compared to non-relapsing patients already 9 months before relapse (*P =* 0.02).

**Discussion:**

Our data indicate that IgG Fc-bisection correlates with long-term treatment outcome, while lower IgG Fc-fucosylation and sialylation associate with impending relapse. Overall, our study replicated the previously published reduction in total IgG Fc-sialylation at the time of relapse, but showed additionally that its onset precedes relapse. Furthermore, our findings on IgG fucosylation and bisection are entirely new. All these IgG Fc-glycosylation features may have the potential to predict a relapse either independently or in combination with known risk factors, such as a rise in ANCA titre.

## 1 Introduction

Anti-neutrophil cytoplasmic antibody (ANCA) – associated vasculitis (AAV) is a group of autoimmune inflammatory diseases comprising microscopic polyangiitis (MPA), eosinophilic granulomatosis with polyangiitis (EGPA) and granulomatosis with polyangiitis (GPA). AAV is characterized by necrotizing vasculitis of small to medium-sized vessels (1). The worldwide annual incidence rate of these diseases is estimated at 10-30 per million, with a prevalence of 50-420 per million (2). AAV is a disease that shifts between phases of remission and relapse. Active AAV can be brought into remission with strong-acting immunosuppressive medication. Once remission is achieved, medication is tapered, and maintenance therapy is started to prevent disease relapse. In general, maintenance therapy is stopped after 1-2 years. Disease relapses occur in 50% of patients within 5 years after the original diagnosis. Disease remission is defined by the absence of active disease manifestations. In contrast, patients are considered to relapse when symptoms of active vasculitis reoccur or new onset of the disease appears (3).

A hallmark of this vascular disease is the presence of pathogenic ANCAs targeting cytoplasmic antigens expressed in the primary granules of neutrophils, either proteinase 3 (PR3) or myeloperoxidase (MPO). PR3-ANCAs are strongly associated with GPA, while MPO-ANCAs coincide strongly with MPA (4, 5). ANCAs are mainly of the IgG isotype, predominantly of the IgG1 and IgG4 subclass, and the pathogenic potential of ANCAs has been repeatedly demonstrated in various animal models (6, 7). Autoantibodies contribute to the development of AAV through the excessive activation of cytokine-primed neutrophils, accompanied by the release of reactive oxygen species, proteolytic enzymes, and neutrophil extracellular trap formation, leading to endothelial damage (8). ANCAs activate by co-ligating antigens and Fc gamma receptors (FcγRs). The Fab portion of ANCAs binds PR3 or MPO antigens, translocated from the cytoplasmic granules to the cell surface, and the crystallizable fragment (Fc) portion binds FcγRs, FcγRIIa, and/or FcγIIIb (9). IgG subclass or a post-translational modification could potentially influence the activation of the inflammatory mechanism, as they strongly modulate Fc-FcγR interaction.

**Figure 1:**
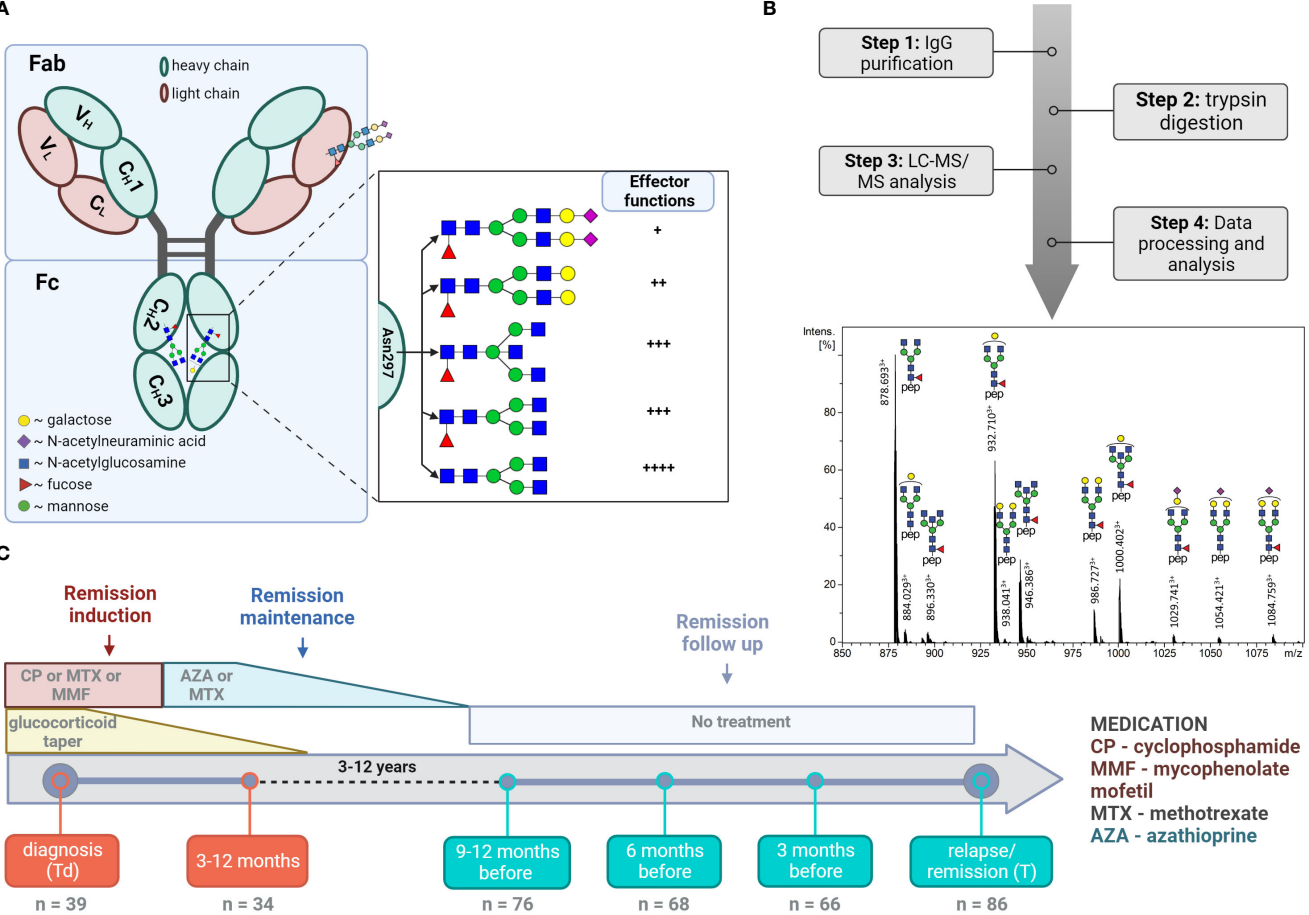
LC-MS analysis of total IgG Fc-glycopeptides. **(A)** Schematic representation of IgG N-glycosylation. The left side depicts IgG with the heavy γ chains (H) and light chains (L) composed of constant (C_H_1, C_H_2, C_H_3, C_L_) and variable domains (V_H_, V_L_). The right side depicts the glycan features covalently attached to Asparagine 297 (Asn 297) and their implications on subsequent cellular effector functions mediated by binding to Fc receptors. **(B)** Schematic workflow with a representative LC-MS summed mass spectrum of IgG1 tryptic glycopeptides obtained from a 60 year-old female at the point of relapse. **(C)** Timeline of the study cohort. The analysed time points and number of samples analysed at each time point are specified. The major treatment regime applied in the current study is shown (10).

Human IgG carries a pair of oligosaccharides attached to Asn297 in C_H_2 domain of the Fc portion. Typical IgG Fc N-glycans consist of a heptasaccharide N-glycan core (four *N*-acetylglucosamine and three mannose residues), which can be elongated with various monosaccharides, such as galactose, sialic acid, fucose, and bisecting N-acetylglucosamine (see **Figure 1**). Glycosylation of the Fc region, along with IgG subclass, has been found to modulate IgG effector functions (11). Altered IgG Fc-glycosylation has been demonstrated in various autoimmune diseases, including AAV (12, 13). Moreover, IgG Fc-glycosylation has been observed to reflect the inflammation status of the body, correlate with disease activity and predict clinical outcomes in many of them (13).

Early diagnosis and accurate disease relapse prediction can greatly contribute to the prevention of disease-related organ damage and mortality in patients with AAV. Patient stratification aims to identify patients with a low and high risk of relapse to guide maintenance therapy, thereby reducing incidences of relapses, while minimizing treatment-related side effects (14). Nowadays, diagnosis and relapse prediction are based on clinical manifestations, histology, and the serial measurements of ANCA titre (15). The detection of a rise in ANCA titre during remission may predict future disease flares. However, many relapses may occur without a preceding ANCA rise and in some cases, a rise in ANCA titre is not followed by a relapse. On top of that, many differences in the definition of a rise in ANCA exist, influencing the usefulness of serial ANCA measurement and making comparisons between studies difficult (16). Thus, while the pathogenic potential of ANCAs to activate the innate immune system, leading to vasculitic damage in AAV, is undisputable, an ANCA rise by itself has a modest value for predicting relapses (17). Therefore, it is important to study other factors involved in the final autoimmune response to arrive at a multi-marker panel with a higher predictive value. IgG Fc-glycosylation status of the antibodies is a promising candidate because of its crucial impact on the key IgG effector functions.

Previous studies have investigated the glycosylation pattern of IgG and demonstrated significantly lower levels of IgG galactosylation in patients with PR3-ANCA or MPO-ANCA compared to healthy individuals (18–20). Moreover, in GPA patients, total IgG galactosylation has been shown to negatively correlate with the disease activity (12). Kemna et al. have revealed that a low level of galactosylation and sialylation in total IgG predicts relapse (21). Interestingly, glycosylation of the total serum antibody pool was a more powerful predictor than that of the ANCA antibodies themselves. These results suggest a potential for the total IgG Fc-glycosylation pattern as a prognostic marker to identify patients more prone to future relapse. Targeting such patients with more aggressive therapy could lead to a better disease prognosis while avoiding over-treatment of patients with a lower risk. Though previous studies indicated time-dependent and intra-individual differences, the longitudinal IgG Fc-glycosylation changes and their clinical relevance are insufficiently mapped, limiting the evaluation as a prognostic marker. Thus, the present work aimed to extensively characterise longitudinal changes of IgG Fc-glycosylation in a single-center AAV patient cohort by profiling changes in N-linked Fc glycans of total IgG in individual AAV patients by mass spectrometry.

## 2 Materials and methods

### 2.1 Patients classification

For this study, 96 patients diagnosed with ANCA-AAV between 2003 and 2016 at the University Medical Groningen, were considered for inclusion. Patients were diagnosed with GPA or MPA according to the Chapel Hill Consensus Conference criteria (1), and categorized as PR3-ANCA or MPO-ANCA based on ANCA-specificity determined using antigen specific enzyme-linked immunosorbent analysis. Disease activity states were characterized in accordance with the European League Against Rheumatism definitions. Clinical remission of the disease was defined as the absence of active vasculitic disease manifestations, with or without taking immunosuppressive therapy. In contrast, relapse was defined as the re-occurrence of symptoms or new onset of disease activity requiring augmentation of immunosuppressive therapy (3). The severity of symptoms during the onset or reoccurrence of the disease was classified as “moderate” or “severe” based on the potential threat to organs or life (3). Persistent positive or negative patients are defined as individuals who consistently maintain positive or negative status for ANCAs. Those initially positive, but transitioning to ANCA negativity following treatment and maintaining this negative status thereafter are classified as ANCA negative to positive patients.

All information on demographics, disease characteristics, treatment, as well as clinical outcome were collected into patient’s records according to the Dutch law on Medical Treatment Act (WGBO), the Persona Data Protection Act (Wbp), and the Code of Conduct for Health Research (Federa). Ethical approval for the study was obtained from the local Medical Ethical Committee of the University of Medical Center Groningen.

### 2.2 Measurement of ANCA and detection of an ANCA rise

ANCA-associated vasculitis patients were generally seen every 3 months for 2 to 3 years. At every follow-up visit, symptoms were evaluated and blood was drawn. After conversion to serum, PR3-ANCA or MPO-ANCA titres were quantified by fluorescent-enzyme immune assay on a Phadia ImmunoCAP 250 analyzer using EliA PR3S and EliA MPOS wells (Thermo Fisher Scientific, Waltham, MA) (22). A rise in ANCA titre was ascertained if the PR3- or MPO-ANCA level increased by at least 125% over a period of six months preceding a clinical relapse or time-matched remission.

### 2.3 Antibody affinity enrichment and protease treatment

Total IgG (IgG1, IgG2, IgG3, and IgG4) was affinity-purified and digested with trypsin in 96-well filter plates (0.7 mL wells, PE frit, Orochem, Naperville, IL) as described before (23). Briefly, plasma samples (2 µL) were diluted with 1xPBS (1:20 v/v) and applied on Protein G affinity beads (2 µL; GE Healthcare, Uppsala, Sweden) for 1 hour with agitation. Captured IgGs were washed with 1xPBS and water (3 x 200 µL), eluted with 100 mM FA (100 µL) and dried in a centrifugal vacuum concentrator (Martin Christ Gefriertrocknungsanlagen GmbH, Osterode am Harz, Germany) at 50°C for approximately 2.5 h. Next, dried total IgG was dissolved by adding 0.25 mg/mL TPCK-treated trypsin (40 µL) in 25 mM ammonium bicarbonate buffer (pH 8.0) and digested for 18 h at 37°C. Obtained IgG glycopeptides were stored at -20°C until the measurement.

### 2.4 Liquid chromatography - mass spectrometry of immunoglobulin G glycopeptides

The tryptic IgG glycopeptide separation was performed by an Ultimate 3000 HPLC nano-LC system (Thermo Fisher Scientific) as described previously (23). (Glyco-)peptides (0.25 µL) were concentrated on the trapping column (Acclaim PepMap100 C18, 5 mm x 300 µm i.d., Thermo Fisher Scientific) and separated on the analytical column (nanoEase M/Z Peptide BEH C18, 100 mm x 75 µm i.d., 1.7 µm, 130 Å, 1/PK, Waters) in a linear gradient from 3% to 50% solvent B (95% acetonitrile) in 5.5 min. The separation system was coupled online by electrospray ionization (ESI) to a Maxis Impact HD quadrupole time-of-flight MS instrument (Bruker Daltonics, Bremen, Germany). The sample was nebulized and ionized in ion positive mode in a CaptiveSpray ESI source assisted by ACN-doped nitrogen gas from the nanoBooster (Bruker Daltonics). Mass spectra were acquired within a mass range *m/z* 550-1800 at a frequency of 1 Hz.

### 2.5 Data analysis

The raw glycoproteomic LC-MS data were first manually inspected with Data Analysis (version 5.0; Bruker Daltonics). The separation method resulted in three subclass-specific IgG N-glycopeptide moieties that were assigned based on well-established migration orders of tryptic Fc-glycopeptides in reversed phase liquid chromatography, such as IgG1, IgG4 and indistinguishable IgG2 and IgG3 (IgG1: EEQYNSTYR, IgG2/3: EEQFNSTFR, IgG4: EEQFNSTYR) (24, 25). Next, the LC-MS datasets were converted into the mzXML file format using MSConvert (ProteoWizard 3.0 suite). Each dataset was then automatically aligned, calibrated, and extracted using the software package LaCyTools as described previously (26), with minor modifications in processing parameters (see **Table S1** for details). For targeted data extraction, a list of pre-defined analytes with theoretical *m/z* values was compiled on the basis of the literature (27) and completed by the manual annotation of summed spectra per patient group. After signal extraction, low-quality spectra with a total intensity or number of glycopeptides below a lower or above an upper threshold were discarded per subclass from further analysis. Lastly, analytes were selected based on quality control parameters provided by LaCyTools, including mass accuracy (within a ± 20 ppm range), signal to noise ratio (> 9), and isotopic pattern quality score (<0.2). For all 37 glycopeptide species passing the analyte inclusion criteria (**Table S2**, 18 IgG1 glycoforms, 11 IgG2 glycoforms, 8 IgG4 glycoforms), relative intensities within each IgG subclass were obtained by integrating and summing signal intensities of doubly- and triply-protonated glycan species followed by normalization to the total signal intensity per IgG subclass. From the individual glycopeptides, four glycosylation traits were calculated (**Table S3**): galactosylation, fucosylation, bisection, and sialylation. Due to the overlap of *m/z* values of the minor afucosylated IgG4 glycopeptides with tailing and more abundant IgG1 fucosylated glycopeptide peaks, fucosylation levels for IgG4 were not quantified.

### 2.6 Statistical analysis

All statistical analysis and data visualization were performed using R Statistical Software (version 4.1.3; R Core Team 2022) and RStudio (version 1.4.1717; RStudio, Boston, MA). Both age and sex have been shown to influence IgG glycosylation (28). To focus on longitudinal changes and reduce the impact of such inter-individual differences, glycosylation was normalized within each individual to the final timepoint, being relapse or time-matched remission. Delta (Δ) values were calculated by subtracting IgG glycosylation levels at the time of relapse or time-matched remission – T(Rel) – from each time point of follow-up – Tx –, according to Formula 1. Pairwise comparison with a non-parametric Wilcoxon rank-sum test between relapsing and non-relapsing patients was used to determine differences in Δglycosylation traits and in relative intensities of the glycosylation traits in patients with an ANCA rise across all time points. A two-sided p-value was considered significant when below 0.05. For multiple testing correction, a 5% false discovery rate was applied throughout using the Benjamini-Hochberg method. An ANOVA F-test, comparing glm models with and without ANCA positivity, was used to assess confounding effects of this feature. The Wilcoxon sign-ranked test was used to test changes within Δglycosylation over time. Longitudinal glycosylation trait data were evaluated by means of Restricted Maximum Likelihood with the *post-hoc* Tukey test. Spearman’s correlation was used to explore the association between IgG1, IgG2/3, and IgG4 glycosylation. The average coefficient of variation (CV) of the relative intensities was calculated in 94 replicate measurements of a pooled sample to further assess the quality of the data (**Figure S1**). A spread was measured by means of the interquartile range and the Kolmogorov-Smirnov test was used to determine differences in the distribution of the data.


(1) $$\Delta =T_{x}-T\left(Rel\right)$$

## 3 Results

In this study, we explored changes in total serum IgG Fc-glycosylation over the course of AAV treatment (see **Figure 1**). To this end, IgG was affinity-purified from 410 longitudinal samples collected from 89 individual patients. Of these 89 patients, 63 were PR3-positive and 26 were MPO-positive. The patient demographics and comprehensive cohort characteristics are presented in **Table 1**. IgG Fc-glycosylation profiles were determined by LC-MS glycopeptide analysis individually for IgG1 and IgG4 (tryptic peptides IgG1: EEQYNSTYR, IgG4: EEQFNSTYR), as well as collectively for IgG2 and IgG3 (shared peptide sequence EEQFNSTFR). LC-MS analysis allowed the identification of 18, 11, and 8 Fc-glycopeptides for IgG1, IgG2/3, and IgG4, respectively (**Table S2**). 94 replicates of a patient plasma pool demonstrated low technical variability. Average coefficient of variation (CV) was 6.5%, 7.2%, and 6.4% for IgG1, IgG2/3, and IgG4 glycopeptides, respectively (**Figure S1**). To reflect the various enzymatic steps involved in glycosylation, derived glycosylation traits, including galactosylation, sialylation, fucosylation and bisection, were calculated from individual glycoforms (**Table S3**). There was a strong correlation between derived traits of IgG1 and IgG2/3, as well as IgG4 in the study patients (**Figure S2**), indicating that all IgG subclasses follow the same general trend in glycosylation changes. Therefore, IgG2/3 and IgG4 data are shown in the Supplemental data.

**Table 1:** Patient characteristics of the study group.

	Total (n = 89)			
	MPO-ANCA		PR3-ANCA	
	**Remission** **(n = 15)**	**Relapse** **(n = 11)**	**Remission** **(n = 42)**	**Relapse** **(n = 21)**
Demographics				
Age (average in years + SD)	67 (17)	67 (12)	61 (12)	59 (15)
Male	9 (60%)	6 (55%)	16 (38%)	9 (43%)
Disease subtype				
GPA	0 (0%)	0 (0%)	42 (100%)	21 (100%)
MPA	15 (100%)	11 (100%)	0 (0%)	0 (0%)
ANCA^a^				
Increase in titre	0 (0%)	3 (25%)	8 (20%)	13 (62%)
ANCA positivity				
Persistent ANCA positive	2 (16%)	4 (36%)	26 (62%)	18 (86%)
Persistent ANCA negative	11 (68%)	4 (21%)	9 (21%)	2 (9%)
Inconclusive data	2 (16%)	2 (43%)	7 (17%)	1 (5%)
Severity^b^				
Moderate	2 (13%)	4 (36%)	3 (7%)	1 (5%)
Severe	13 (87%)	5 (46%)	32 (76%)	19 (90%)
No information	–	2 (18%)	7 (7%)	1 (5%)
Induction treatment^c^				
Glucocorticoid therapy (GC)	2 (13%)	0 (0%)	4 (10%)	–
Cyclophosphamide + GC	10 (67%)	5 (46%)	29 (69%)	13 (62%)
Mofetil mycophenolate (MMF) + GC	3 (20%)	1 (9%)	3 (7%)	6 (28%)
Methotrexate (MTX) + GC	–	2 (18%)	–	1 (5%)
Rituximab (RTX) + GC	–	1 (9%)	–	–
No information		2 (18%)	6 (14%)	1 (5%)
Organ involvement^d^				
Ear, nose, throat	2 (13%)	1 (9%)	32 (76%)	19 (90%)
Lung	7 (47%)	3 (27%)	22 (52%)	20 (95%)
Renal	13 (87%)	7 (64%)	22 (52%)	15 (71%)
No information		3 (27%)	6 (14%)	1 (5%)

SD, standard deviation.

a A rise in ANCA titre preceding relapse determined using a highly sensitive capture enzyme-linked immunosorbent assay (ELISA).

b Severity of symptoms at previous disease flare (diagnosis or any previous relapse) (3).

c Medication scheme used to induce remission.

d Organ-threatening manifestation of disease at previous disease flare (diagnosis or any previous relapse).

### 3.1 Changes in IgG Fc-glycosylation

**Figure 2:**
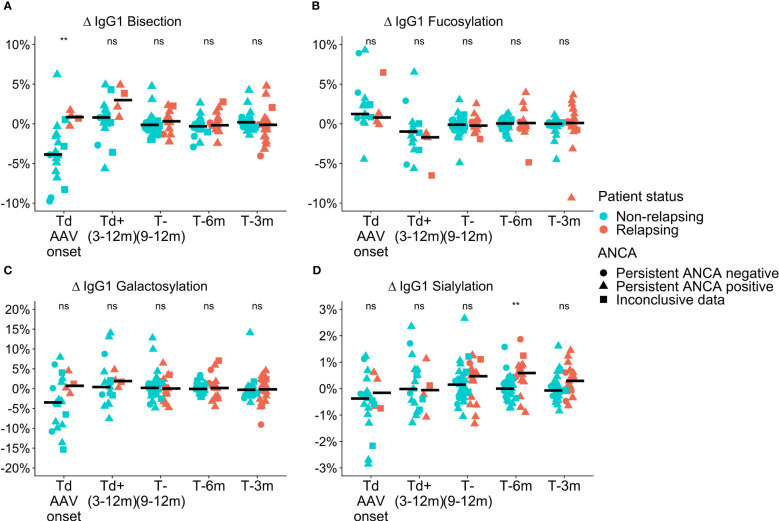
Cross-sectional differences in IgG1 glycosylation features for GPA-PR3-ANCA patients. Differences compared to the point of relapse or time-matched remission are displayed as Δ values, separately for patients who either relapsed (orange) or stayed in remission (green). The shapes represent patients who are persistently ANCA negative (circle), persistently ANCA positive (triangle), or show inconclusive results (square). Time points range from disease diagnosis (Td AAV onset), and 3-12 months after diagnosis (Td+(3-12m)), to 9-12 months (T-(9-12m)), 6 months (T-6m), and 3 months (T-3m) before relapse or time-matched during remission.Panels show the four investigated glycosylation features, namely **(A)** Bisection, **(B)** Fucosylation, **(C)** Galactosylation, and **(D)** Sialylation. Median values are indicated by black horizontal bars. A Wilcoxon signed-rank test was used to compare IgG1 glycosylation traits. Findings are indicated by two asterisks **, (p< 0.005); ns, not significant (see also **Table S4**). An ANOVA F-test showed that these findings were independent of ANCA positivity.

Differences in total IgG Fc-glycosylation relative to the timepoint of relapse or time-matched remission (Δ values) were observed between relapsing patients and patients in remission in GPA with PR3-ANCA or MPA with MPO-ANCA. At the time of diagnosis in GPA-PR3-ANCA patients, ΔIgG1 bisection of relapsing patients was higher than in non-relapsing patients (IgG1 0.8% vs. -3.9%; *P =* 0.004, **Table S4**; **Figure 2A**). A similar trend in medians was also found to a lower extent 9 to 12 months before relapse. These differences were not confounded by ANCA positivity as no significant differences between the glm models were observed. Bisection increased by 4.7% upon initial treatment in non-relapsing PR3-ANCA patients (**Figure S3D**). For these patients, the spread in Δbisection ([IQR = Q3 – Q1] 4.42 vs. 1.53, *P =* 0.0003) appeared to be higher at diagnosis (Td) when compared to an early treatment phase, 3 to 12 months after diagnosis (Td+(3-12m)) (**Table S5**). Glycosylation data on IgG2/3 and IgG4 data are shown in the **Figure S4**.

ΔIgG1 sialyation of the GPA-PR3-ANCA relapsing patients was increased in the year leading up to the relapse and differed from non-relapsing patients at 6 months (and potentially at 3 months) before relapse (IgG1 0.6% vs. 0.0%; *P =* 0.002, 0.4% vs. -0.1%; *P =* 0.044, **Table S4**; **Figure 2D**). Also, these findings were not confounded by ANCA positivity. Moreover, the longitudinal analysis revealed a decrease in ΔIgG1 sialylation over 6 months preceding relapse (*P =* 0.0004, **Figure S3A**), whereas ΔIgG1 sialylation in non-relapsing patients remained constant. No differences between relapsing and non-relapsing GPA-PR3-ANCA patients were observed for Δfucosylation and Δgalactosylation (**Figures 2B, C**).

Sialylation and galactosylation of both IgG1 and IgG2/3 showed a negative correlation with the level of PR3-ANCAs (IU/mL) in plasma of relapsing patients (**Figures S5A, B, E, F**). In contrast, the levels of bisection of both IgG1 and IgG2/3 showed a positive relationship with the PR3-ANCA concentration in relapsing GPA patients (**Figures S5D, H**). Regression analysis of IgG4 galactosylation as well as the sialylation versus PR3-ANCA titre did not show an ANCA titre dependence (**Figures S5I, J**).

Conversely, relapsing MPA-MPO-ANCA patients showed a trend towards a more pronounced lowering in Δbisection at diagnosis compared to non-relapsing patients (IgG1 -3.5% vs.-1.05% **Table S6**; **Figure S6A**). Subsequently, relapsing MPA-MPO-ANCA patients experienced an increase in Δbisection by 3.9% up to the second follow-up (**Figure S7**; *P =* 0.002). Notably, the Δsialylation effects are not observed for the MPA-MPO-ANCA group (**Figures S6D**). Δfucosylation, as well as Δgalactosylation of the IgG Fc glycoforms, did not differ during the course of disease treatment between relapsing and non-relapsing MPA-MPO-ANCA patients (**Figures S6B, C**).

There were no correlations between IgG1, IgG2/3, and IgG4 glycosylation traits and MPO-ANCA titre MPA-MPO-ANCA patients (**Figure S8**).

### 3.2 IgG Fc-glycosylation changes associate with an ANCA rise

**Figure 3:**
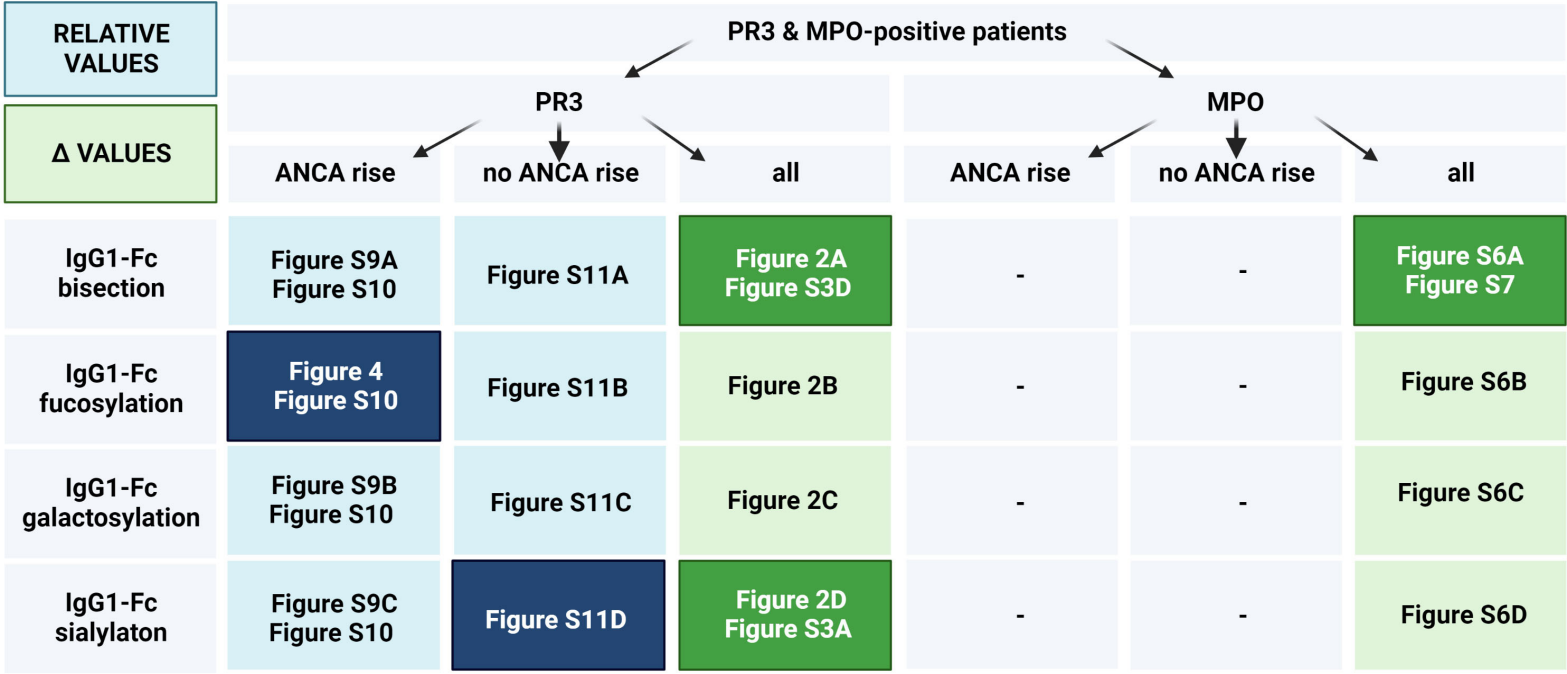
Figure layout of the IgG glycosylation analyses for GPA-PR3-ANCA and MPA-MPO-ANCA patients with and without an ANCA rise. Cells are colored based on the IgG glycosylation values (Blue rectangles correspond to the analysis based on relative levels of IgG glycosylation, while green rectangles correspond to Δ values). Dark colors indicate figures with statistically significant findings, longitudinally (**Figures S3, 7, 10**), or between relapsing and non-relapsing patients (other figures).

**Figure 4:**
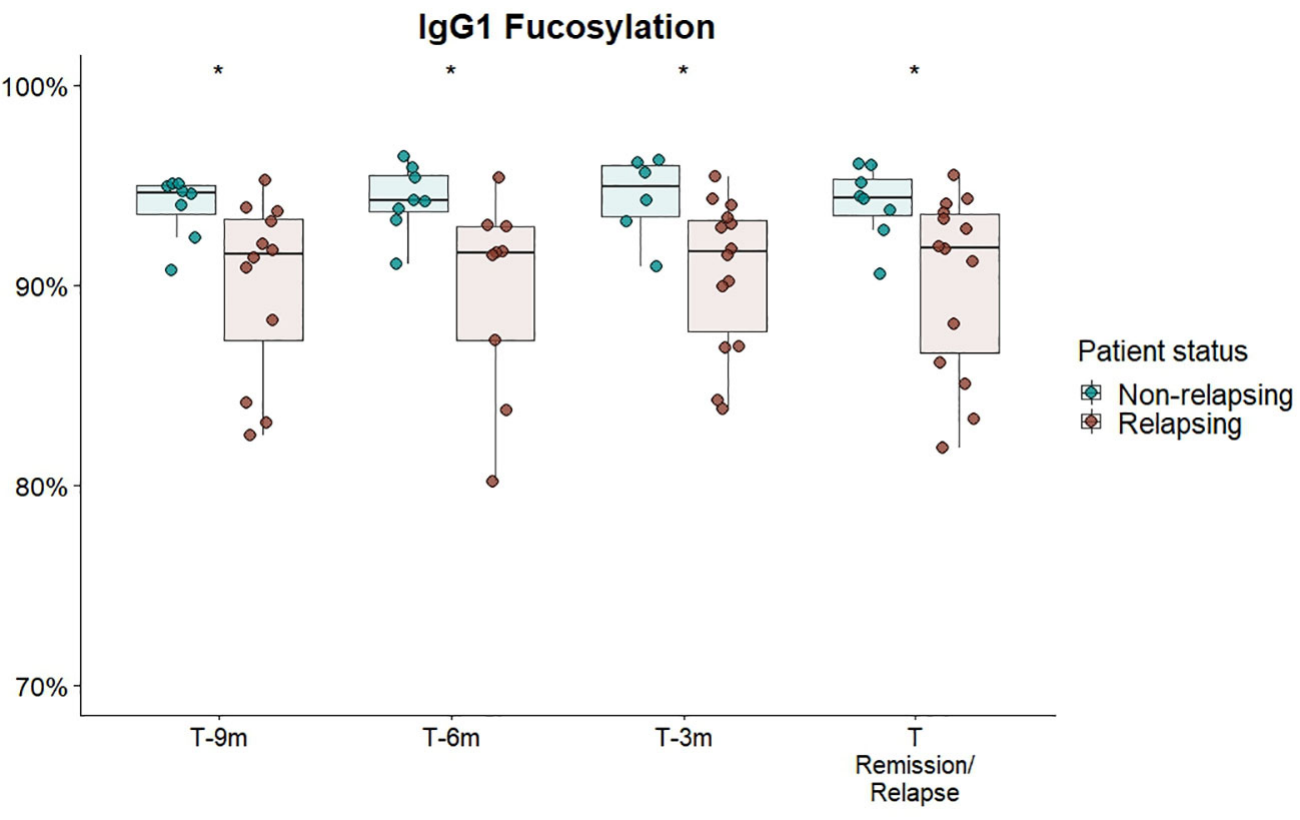
Cross-sectional differences in IgG1 Fc-fucosylation for GPA-PR3-ANCA patients with an ANCA rise. Total serum IgG1 fucosylation of GPA-PR3-ANCA patients who either relapse (brown) or stayed in remission (green), was compared at 9 months, 6 months before, 3 month before and at the time of relapse or time-matched point during remission. A Wilcoxon signed-rank test was used to compare IgG1 glycopeptide traits. * T-6m is a finding after multiple testing correction (p=0.011; see **Table S7**). However, since there is an according trend in all timepoints (p=0.02), the probability of the trends being false negative findings (should they be excluded) is more than 99.99%.

We separately analysed a subset of GPA-PR3-ANCA patients who experienced an ANCA rise that was defined as at least 125% elevation in ANCA level relative to the previous titre measurement (see **Figure 3**). Only in the subset of GPA-PR3-ANCA patients with an ANCA rise, relapsing patients showed a lower fucosylation compared to patients in remission in the year before relapse (T-9 months 91.6% vs. 94.6%, *P =* 0.02; T-6 months 91.6% vs. 94.2%, *P =* 0.01; T-3 months 91.7% vs. 95.0%, *P =* 0.02) and at the point of relapse or time-matched during remission (91.9% vs. 94.4% *P =* 0.02) (**Figure 4**; **Table S7**). Neither bisection, galactosylation, nor sialylation differed between patients relapsing or in remission (**Figures S9A–C**). Longitudinal changes were not appreciated at all (**Figure S10**).

Interestingly, the IgG fucosylation difference in GPA-PR3-ANCA patients between relapsing patients and patients in remission was limited to patients with an ANCA rise. It did not emerge in patients without an ANCA rise (**Figure S11B**) nor when looking at the whole GPA-PR3-ANCA patient group (**Figure 2B**), even though patients experiencing an ANCA rise were in the majority.

## 4 Discussion

GPA and MPA are distinct disease subtypes, but are highly associated with PR3- and MPO-ANCA, respectively. Previous studies additionally indicated that PR3-ANCA and MPO-ANCA-associated diseases are significantly different (29–31). Consequently, we analysed data from GPA-PR3-ANCA and MPA-MPO-ANCA patients separately throughout this investigation. Our study indicates distinct cross-sectional differences in IgG Fc-glycosylation between GPA-PR3-ANCA and MPA-MPO-ANCA patients. Unfortunately, we are not able to determine whether disease subtype or ANCA-type is dominant in these differences. Comparing relapsing with non-relapsing patients, GPA-PR3-ANCA patients exhibited a higher degree of ΔIgG1 bisection at diagnosis, while MPA-MPO-ANCA patients showed a trend towards a lower degree of ΔIgG1 bisection if they would relapse (**Figures 2**, **S6**). The trend in MPA-MPO-ANCA patients is confirmed by an increase in ΔIgG1 bisection from diagnosis to 9 to 12-month follow-up in relapsing patients which is not observed in non-relapsing patients (**Figure S7**). ΔIgG1 sialylation only associated with relapse in GPA-PR3-ANCA, but not in MPA-MPO-ANCA patients (**Figures 2**, **S6**).

In our study, the focus on total serum IgG Fc-glycosylation is justified by the stronger associations with relapse, as compared to ANCA glycosylation, demonstrated previously (21). A cross-sectional comparison revealed that ΔIgG1 bisection and ΔIgG1 sialylation of total serum IgG Fc N-glycans differed between GPA-PR3-ANCA patients who relapsed and those who stayed in remission (**Figure 2**). Interestingly, while bisection differed mainly at the time of diagnosis, sialylation only differed in the year leading up to relapse. Accordingly, longitudinal analysis showed that a decrease in sialylation precedes relapse in GPA-PR3-ANCA patients, while there are no changes in sialylation for non-relapsing patients. (**Figure S3**). Similarly, Kemna et al. showed a reduction in sialylation at relapse compared to an ANCA-rise time point, whereas patients who stayed in remission had unchanged sialylation levels (21). Our analysis replicates this finding, but additionally demonstrates that the reduction starts before the reoccurrence of symptoms. Moreover, sialylation negatively correlated with the level of pathogenic PR3-ANCA titre in relapsing GPA patients (**Figure S5**). Reduced sialylation indicates a general inflammatory environment which may be the cause and/or effect of enhanced antibody production (32). Thus, a longitudinal reduction in total IgG Fc-sialylation may be a potential clinical marker of relapse risk. Sialylation on IgG has been reported to have anti-inflammatory potential through a shift in IgG conformation, preventing the antibody from binding to activating type I Fc receptors and C1q protein and enabling interaction with type II Fc receptors DC-SIGN and CD23, which in turn induces the loss of the cytotoxic capability and FcγR-mediated anti-inflammatory activities, respectively (33–35). Lower amounts of sialylated IgGs during inflammation have further been correlated with enhanced activity of autoimmune diseases, including GPA (12, 21). Intriguingly, this potential marker may thus be directly involved in the disease mechanism. It has been suggested that a higher sialylation could counterbalance the autoimmune cascade induced by ANCA, and a subsequent shift towards a lower degree of sialylated IgGs would reduce its protective anti-inflammatory effect, possibly contributing to disease reactivation (21). Therefore, we speculate that IgG Fc-sialylation could be used for monitoring relapse risk in patients with GPA-PR3-AAV. Longitudinal monitoring of the patient’s level of IgG Fc-sialylation and detecting its drop, could aid in initiating appropriate treatment to prevent disease recurrence. Such clinical marker guided preemptive therapy could prevent further damage to the patient’s vasculature. However, more data – ideally with an even higher time-resolution – would be needed to conclude whether sialylation changes occur sufficiently early prior to relapse to be of diagnostic value. We did not observe any differences in IgG Fc-galactosylation, which contradicts previous reports (18, 21). For example, Lardinois et al. showed that terminal galactose was reduced in GPA-PR3-ANCA patients with active disease compared to patients in remission (18). Moreover, Kemna et al. revealed a reduced galactosylation and sialylation in total IgG Fc of relapsing patients compared to non-relapsing patients (21). However, their cohort was focused exclusively on GPA patients with PR3-ANCA experiencing an ANCA rise, which means that our study is likely underpowered regarding this very specific finding. Due to the complexity of AAV, for example in antigen specificity, definitions of an ANCA rise and disease phenotype, differences between studies in terms of study design, clinical cohort characteristics, and the definition of the relapse and an ANCA rise, have to be carefully considered when comparing them and may explain observed differences.

This study identified, for the first time, a persistently lowered IgG Fc-fucosylation as a feature of relapsing GPA-PR3-ANCA patients experiencing an ANCA rise, discriminating them from non-relapsing ones (**Figure 4**). Interestingly, no differences in IgG1 fucosylation were observed for relapsing patients without a preceding rise in the ANCA titre (**Figure S11**). This may indicate differences in pathological mechanisms between AAV patients with and without an ANCA rise. These observations lead us to speculate on the potential utility of differences in IgG Fc-fucosylation as an orthogonal biomarker, enhancing the specificity of ANCA rise in clinical practice for predicting relapse. Importantly, differences in IgG Fc-fucosylation between relapsing and non-relapsing patients can be detected as early as nine months prior relapse if a rise in ANCA titer was also observed. Consequently, an ANCA rise and IgG Fc-fucosylation could potentially be integrated into a multiplex biomarker platform. Due to the limited number of MPA-MPO-ANCA patients, the study was underpowered to detect differences within the MPA-MPO-ANCA vasculitis patient population who experienced an ANCA rise. A predominantly afucosylated IgG response may occur naturally during the B-cell immune responses and has been mostly found for antigen-specific IgG1 against enveloped viruses and intracellular parasites, as well as for IgG1 alloantibodies causing fetal and neonatal alloimmune thrombocytopenia and hemolytic disease of the fetus and newborn (36–38). Since afucosylated IgG1 seem to be specific for membrane-bound antigens (37) and PR3-/MPO-antigens are found on neutrophil plasma membranes in AAV (9, 39), afucosylated IgG responses could be present in AAV. Though other factors will likely contribute to the observed differences in afucosylation, the association with an ANCA rise would suggest that such afucosylated autoantibody responses may be a relevant risk factor for relapse. However, Kemna et al. observed high fucosylation of PR3-ANCA antibodies (21). Thus, the observed afucosylation differences are more likely due to changes in IgGs of other specificities (non-autoantigen specific IgG). The same immune activation, which leads to increased production of PR3-ANCA, could specifically stimulate B cells with a low fucosylation programming to differentiate and produce predominantly afucosylated antibodies. Such preferential co-activation of afucosylated antibody producing cells could, for example, result from the co-localization of antibody producing cells in the spleen (40). Another explanation would be a globally altered regulation of Fc-fucosylation, for example changes in cytokine composition in the B cell microenvironment (41, 42), initiated by an increased activity of inflammatory processes. Non-autoantigen specific IgG might be more affected by these changes, as it already has a higher propensity for afucosylated responses than PR3-ANCA.

IgG Fc-fucosylation has been shown to have a significant impact on IgG effector functions by sterically disturbing the interaction between the Fc region and FcγRIIIa/b, a receptor expressed on various effector cells. Thus, fucosylation may act as a safety switch hampering potentially harmful cellular immune functions (43, 44). It has been shown that ANCAs bind to and activate neutrophils and monocytes by initial Fab binding to MPO or PR3 expressed on the cell surface and subsequent Fc binding to FcγRs (FcγRIIa and FcγRIIIb), which induces neutrophil and monocyte activation leading to endothelial cell death and inflammation (45, 46). The complete removal of the Fc N-glycans has been shown to attenuate FcγR-mediated neutrophil respiratory burst and degranulation whilst reducing the disease progression (47). The importance of the IgG Fc-FcγRIIIb interaction for the disease progression of AAV has also been evidenced by the association of the FcγRIIIb-NA1 allele with the development of severe renal disease (48). Moreover, as reported in studies involving therapeutic antibodies, decreased levels of IgG Fc-fucosylation induced a more rapid inflammatory cytokine release and cell activation (49). As opposed to afucosylation of ANCA IgG, afucosylation of IgG with other specificities (aspecific IgG) will also have anti-inflammatory effects. Monomeric, afucosylated, aspecific IgG will compete with the ANCA immune complexes for FcγRIII-binding. Since monomeric IgG will not activate receptor signaling, this competition should reduce FcγRIII-dependent pro-inflammatory signaling (50). While the lower fucosylation of aspecific IgG could be a bystander effect of differences in general inflammation, it may also affect the disease mechanism of ANCA-AAV by reducing the interaction between PR3-ANCA and activating FcγRs on neutrophils.

We revealed a markedly lower bisection for non-relapsing GPA-PR3-ANCA patients at diagnosis (**Figure 1**). Moreover, the longitudinal analysis demonstrated a 4.7% increase in bisection between diagnosis and first follow-up for non-relapsing GPA-PR3-ANCA patients (**Figure S3**). This is likely due to treatment effects. Interestingly, we found that the cross-sectional differences, as well as longitudinal changes, reversed for MPA-MPO-ANCA patients, where relapsing patients showed a trend towards lower bisection at diagnosis followed by an significant increase in bisection up to the second follow-up (**Figures S6**, **S7**). We hypothesize that the treatment effect observed for bisection could be useful to screen patients for their risk of relapse. However, due to the limited number of available samples for GPA-PR3-ANCA patients at early time points, the discriminative potential of bisection needs further justification. The current understanding of the functional relevance of IgG Fc-bisection is limited. At least for the complement activation and FcγR-mediated effector functions, no significant impact of the observed differences would be expected (51). However, IgG Fc-bisection plays a role in other aspects, such as higher-order protein structure and stability (52).

IgG Fc-glycosylation trades of IgG2/3 subclasses showed a high correlation with IgG1 and consistent effects to those observed for IgG1. This indicates that alteration of IgG Fc-glycosylation is most likely a common event in all IgG subclasses, perhaps as a result of an identical stimulation of IgG-secreting plasma cells.

The limitations of this study include the absence of glycosylation analysis of anti-PR3 and anti-MPO specific antibodies. Ultimately, only a direct analysis of autoantibody glycosylation would confirm or exclude afucosylated ANCA responses in AAV. Importantly, this does not limit the clincal relevance of our study, as total serum IgG Fc-glycosylation has demonstrated superior performance as potential clinical marker (21). Another limitation of the study is the sample collection, where not all 89 AAV patients were sampled on six occasions from diagnosis to relapse or time-matched remission, leading to missing time points and temporal spread of the time points. The statistical analysis of relatively small groups of patients constitutes another limitation of the study. However, this is strongly mitigated by the longitudinal setup of the cohort. The possibility to partially correct for inter-individual differences in the longitudinal analyses and the repeated observation of the same cross-sectional differences at different time points greatly enhance the statistical confidence in our findings. Nonetheless, for novel findings, which are not a validation or extension of previous studies, external validation with a larger cohort would be prudent to evaluate if these glycosylation differences also enable relapse risk stratification.

In conclusion, our study confirmed several IgG Fc-glycosylation features that may have the potential to predict relapse either independently or in combination with known risk factors. We were able to replicate the reduced IgG Fc-sialylation at relapse reported by Kemna et al. Importantly, we have additionally demonstrated that this gradual decrease in IgG Fc-sialylation happens over a period of six months and could be detected before relapse occurs, irrespective of the rise in ANCA titre. Our study has revealed previously unreported associations with IgG Fc-fucosylation and IgG Fc-bisection as well. Namely, increased afucosylation in combination with an ANCA rise correlated with relapse risk. This correlation might originate from and/or contribute to enhanced inflammation in the wake of a rise in ANCA titre which ultimately leads to relapse. Moreover, our results have indicated changes in IgG Fc-bisection that accompany treatment. Bisection thus correlates with long-term treatment outcomes, while fucosylation and sialylation associate with impending relapse. Consequently, all these features might be used to develop novel predictors of relapse risk.

## Data availability statement

The raw data supporting the conclusions of this article will be made available by the authors, without undue reservation.

## Ethics statement

The studies involving humans were approved by Medical Ethical Committee of the University Medical Center Groningen. The studies were conducted in accordance with the local legislation and institutional requirements. The participants provided their written informed consent to participate in this study.

## Author contributions

DF, MW, PH and AR designed the study. IW performed the experiments. IW and DF analyzed the data, and wrote the paper. IW and DF performed the statistical analyses. PH, AR, CS developed the clinical sample cohort. All authors contributed to the article and approved the submitted version.
