# Supplementary material for: Specific IgG glycosylation differences precede relapse in PR3-ANCA associated vasculitis patients with and without ANCA rise
Source: Front Immunol. 2023 Sep 29;14:1214945. doi: 10.3389/fimmu.2023.1214945 (PMC10570725; doi:10.3389/fimmu.2023.1214945)
Supplement: Supplementary file 1 [file DataSheet_1.docx]

Supplementary Material

Specific IgG glycosylation differences precede relapse in PR3-ANCA Associated Vasculitis patients with and without ANCA rise

Iwona Wojcik, Manfred Wuhrer, Peter Heeringa, Coen Stegeman, Abraham Rutgers, David Falck^*^

*** Correspondence:** David Falck: [d.falck@lumc.nl](mailto:d.falck@lumc.nl)

Alternative Corresponding Author: Abraham Rutgers: [a.rutgers@umcg.nl](mailto:a.rutgers@umcg.nl)

# Supplementary Data

## Materials

Ultra-pure deionized water (MQ) was generated by a Purelab Ultra, maintained at 18.2 MΩ (Veolia Water Technologies Netherlands B.V., Ede, The Netherlands). Disodium hydrogen phosphate dihydrate (Na_2_HPO_4_∙2H_2_O), potassium dihydrogen phosphate (KH_2_PO_4_), NaCl, and trifluoroacetic acid (TFA) were purchased from Merck (Darmstadt, Germany). Formic acid (FA), ammonium bicarbonate, and TPCK-treated trypsin from bovine pancreas were obtained from Sigma-Aldrich (Steinheim, Germany). Furthermore, HPLC SupraGradient acetonitrile (ACN) was obtained from Biosolve (Valkenswaard, The Netherlands) and phosphate-buffered saline (PBS) was made in-house, containing 5.7 g/L Na_2_HPO_4_∙2H_2_O, 0.5 g/L KH_2_PO_4_ and 8.5 g/L NaCl.

# Supplementary Figures and Tables

## Supplementary Tables

Supplementary Table 1. LacyTools parameters. Parameters for the automated IgG glycopeptide analysis using LaCyTools software.

| **Parameter Settings** | **Value** |
| --- | --- |
| LaCyTools Version | 1.1.0 |
| Alignment Parameters | |
| Alignment time window | 6.0 s |
| Alignment m/z window | 0.1 |
| Minimal S/N for alignment | 100 |
| Minimal features for alignment | 7 |
| Calibration Parameters | |
| Calibration mass window | 0.3 Da |
| Minimal S/N for calibration | 27 |
| Minimal number of calibrants | 6 |
| Extraction Parameters | |
| Data points per 1 m/z | 100 |
| Extraction m/z window | 0.2 Th |
| Extraction time window | 12 s |
| Minimum charge state | 2 |
| Maximum charge state | 3 |
| Minimum isotopic fraction | 0.95 |
| Background detection window | 10 |

Supplementary Table 2. Compositions of human IgG Fc glycans quantified by nano-LC-ESI-MS and the theoretical *m/z* values corresponding to the most abundant isotopologue of tryptic glycopeptides.

| **Glycan composition** | **Alternative nomenclature** | **Proposed structure** | **IgG1 P01857*** | | **IgG2 P01859*** | | **IgG4 P01861*** | |
| --- | --- | --- | --- | --- | --- | --- | --- | --- |
|  |  |  | **[M+2H]^2+^** | **[M+3H]^3+^** | **[M+2H]^2+^** | **[M+3H]^3+^** | **[M+2H]^2+^** | **[M+3H]^3+^** |
| **H3N4** | G0 | 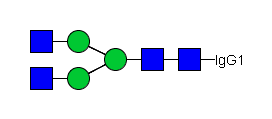 | **1244.50** | **830.00** |  |  |  |  |
| H3N5 | G0N | 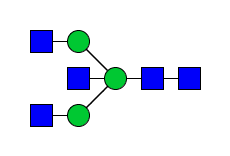 | 1346.04 | 897.69 |  |  |  |  |
| H4N4 | G1 | 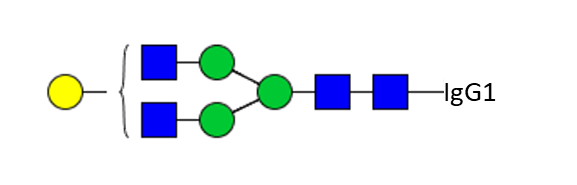 | 1325.52 | 884.02 | 1309.53 | 873.36 |  |  |
| H4N5 | G1N | 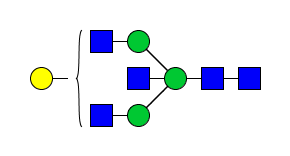 | 1427.06 | 951.71 |  |  |  |  |
| H5N4 | G2 | 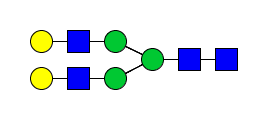 | 1406.55 | 938.04 |  |  |  |  |
| H3N3F1 | G0F-N | 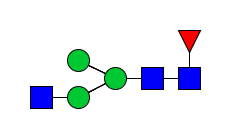 | 1215.99 | 810.99 | 1199.99 | 800.33 | 1207.99 | 805.66 |
| H4N3F1 | G1F-N | 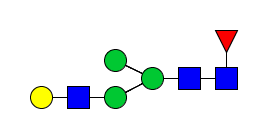 | 1297.01 | 865.01 | 1281.02 | 854.35 |  |  |
| **H3N4F1** | G0F | 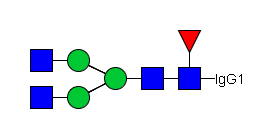 | **1317.53** | **878.69*** | **1301.53** | **868.02*** | **1309.53** | **873.36*** |
| **H4N4F1** | G1F | 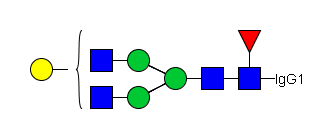 | **1398.55** | **932.70*** | **1382.56** | **922.04*** | **1390.56** | **927.37*** |
| **H5N4F1** | G2F | 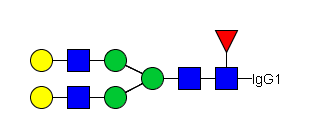 | **1479.58** | **986.72** | **1463.58** | **976.06** | **1471.58** | **981.39** |
| H3N5F1 | G0FN | 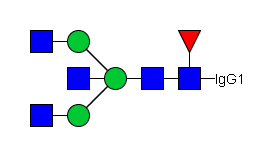 | 1419.07 | 946.38 | 1403.07 | 935.72 | 1411.07 | 941.05 |
| **H4N5F1** | G1FN | 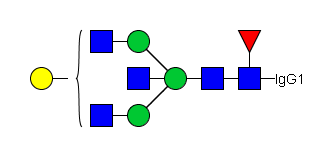 | **1500.09** | **1000.40** | **1484.10** | **989.73** |  |  |
| H5N5F1 | G2FN | 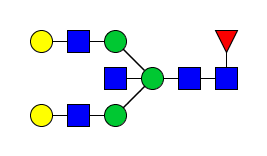 | 1581.12 | 1054.42 | 1565.12 | 1043.75 | 1573.12 | 1049.08 |
| H5N4S1 | G2S | 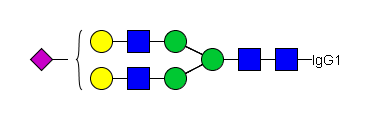 | 1552.10 | 1035.07 |  |  |  |  |
| H4N4F1S1 | G1FS | 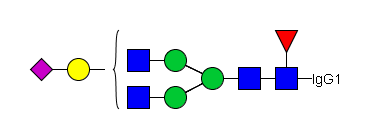 | 1544.10 | 1029.74 | 1528.11 | 1019.07 | 1536.10 | 1024.40 |
| **H5N4F1S1** | G2FS | 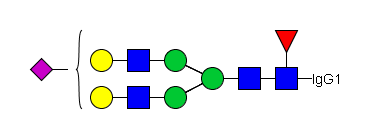 | **1625.13** | **1083.75*** | **1609.13** | **1073.09*** | **1617.13** | **1078.42*** |
| H5N4F1S2 | G2FS2 | 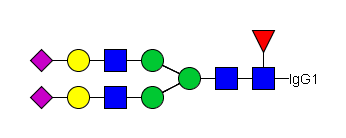 | 1770.67 | 1180.79 |  |  |  |  |
| H5N5F1S1 | G2FNS | 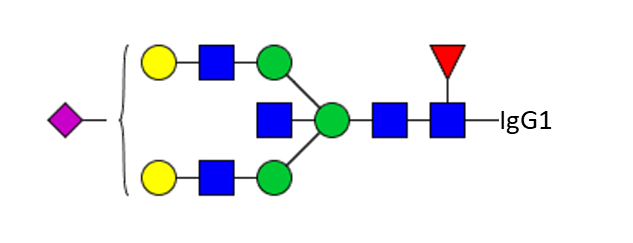 | 1726.67 | 1151.45 |  |  |  |  |

(*) Glycan compositions used for alignment
(bold) Glycan structural feature used for calibration.

Supplementary Table 3. Glycosylation trait calculation. The compositions are placeholders for their respective relative intensities.

| **Glycosylation trait** | **Description** | **Calculation** |
| --- | --- | --- |
| IgG1 Bisection | Bisection on IgG1 | IgG1_H3N5 + IgG1_H3N5F1 + IgG1_H4N5 + IgG1_H4N5F1 + IgG1_H5N5F1 + IgG1_H5N5F1S1 |
| IgG1 Fucosylation | Fucosylation on IgG1 | IgG1_H3N3F1 + IgG1_H4N3F1 + IgG1_H3N4F1 + IgG1_H4N4F1 + IgG1_H3N5F1 + IgG1_H5N4F1 + IgG1_H4N5F1 + IgG1_H4N4F1S1 + IgG1_H5N5F1 + IgG1_H5N4F1S1 + IgG1_H5N5F1S1 + IgG1_H5N4F1S2 |
| IgG1 Galactosylation | Galactosylation per antenna on IgG1 | 1/2 * (IgG1_H4N3F1 + IgG1_H4N4 + IgG1_H4N4F1 + IgG1_H4N5 + IgG1_H4N5F1 + IgG1_H4N4F1S1) + (IgG1_H5N4 + IgG1_H5N4F1 + IgG1_H5N4S1 + IgG1_H5N5F1 + IgG1_H5N4F1S1 + IgG1_H5N5F1S1 + IgG1_H5N4F1S2) |
| IgG1 Sialylation | Sialylation per antenna on IgG1 | 1/2 * (IgG1_H4N4F1S1 + IgG1_H5N4S1 + IgG1_H5N4F1S1 + IgG1_H5N5F1S1) + (IgG1_H5N4F1S2) |
| IgG2/3 Bisection | Bisection on IgG2/3 | IgG2/3_H3N5F1 + IgG2/3_H4N5F1 + IgG2/3_H5N5F1 |
| IgG2/3 Fucosylation | Fucosylation on IgG2/3 | IgG2/3_H3N3F1 + IgG2/3_H4N3F1 + IgG2/3_H3N4F1 + IgG2/3_H4N4F1 + IgG2/3_H3N5F1 + IgG2/3_H5N4F1 + IgG2/3_H4N5F1 + IgG2/3_H4N4F1S1 + IgG2/3_H5N5F1 + IgG2/3_H5N4F1S1 |
| IgG2/3 Galactosylation | Galactosylation per antenna of diantennary glycans on IgG2/3 | 1/2 * (IgG2/3­_H4N3F1 + IgG2/3_H4N4 + IgG2/3_H4N4F1 + IgG2/3_H4N5F1 + IgG2/3_H4N4F1S1) + IgG2/3_H5N4F1 + IgG2/3_H5N5F1 + IgG2/3_H5N4F1S1 |
| IgG2/3 Sialylation | Sialylation per antenna of diantennary glycans on IgG2/3 | 1/2 * (IgG2/3_H4N4F1S1 + IgG2/3_H5N4F1S1) |
| IgG4 Bisection | Bisection on IgG4 | IgG4_H3N5F1+IgG4_H5N5F1 |
| IgG4 Galactosylation | Galactosylation per antenna of diantennary glycans on IgG4 | 1/2 * (IgG4_H4N4F1 + IgG4_H4N4F1S1) + (IgG4_H5N4F1 + IgG4_H5N5F1 + IgG4_H5N4F1S1) |
| IgG4 Sialylation | Sialylation per antenna of diantennary glycans on IgG4 | 1/2 * (IgG4_H4N4F1S1 + IgG4_H5N4F1S1) |

## Supplementary Figures


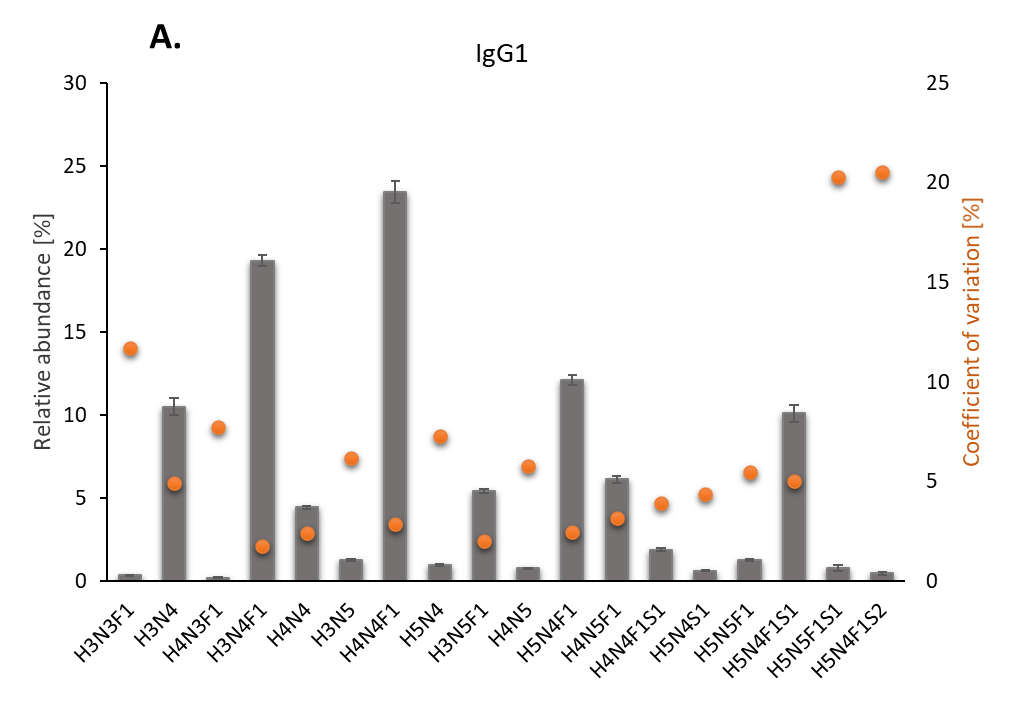


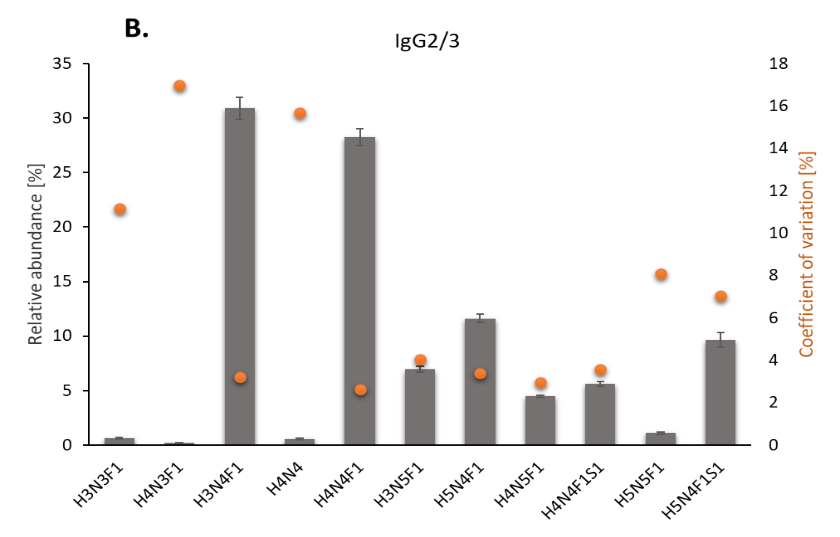


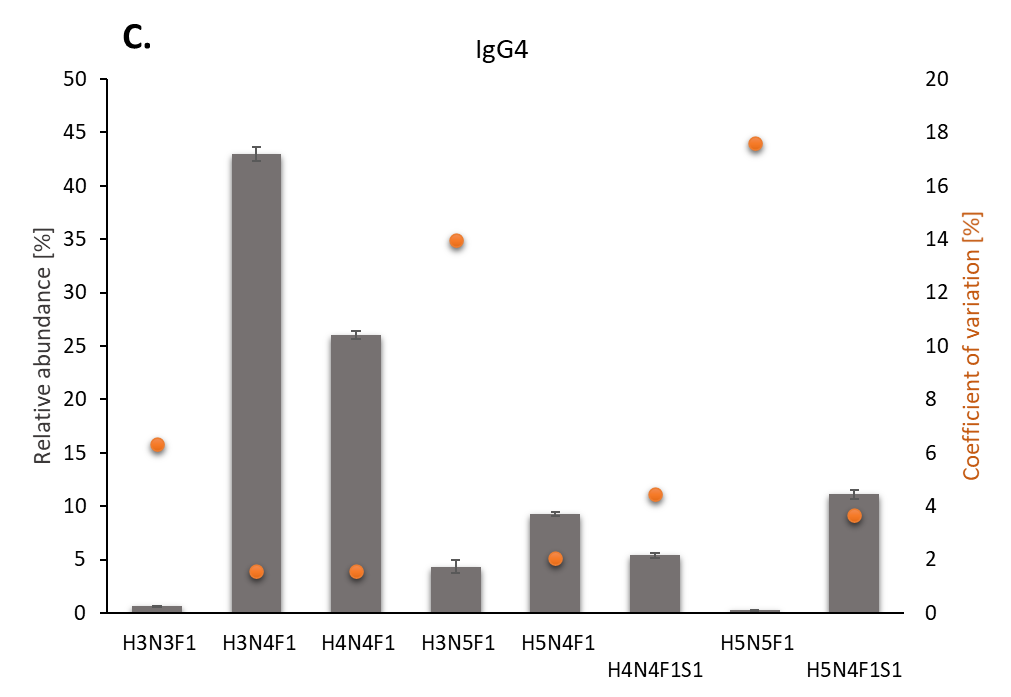


Supplementary Figure 1. Technical variability. Average and coefficient of variation of relative abundances of (**A**) IgG1, (**B**) IgG2/3 and **(C**) IgG4 glycopeptides from 94 replicates of a pooled plasma sample. A median coefficient of variation of 6.5%, 7.2% and 6.4% was observed for IgG1, IgG2/3 and IgG4, respectively. The error bars represent the standard deviation of the mean.


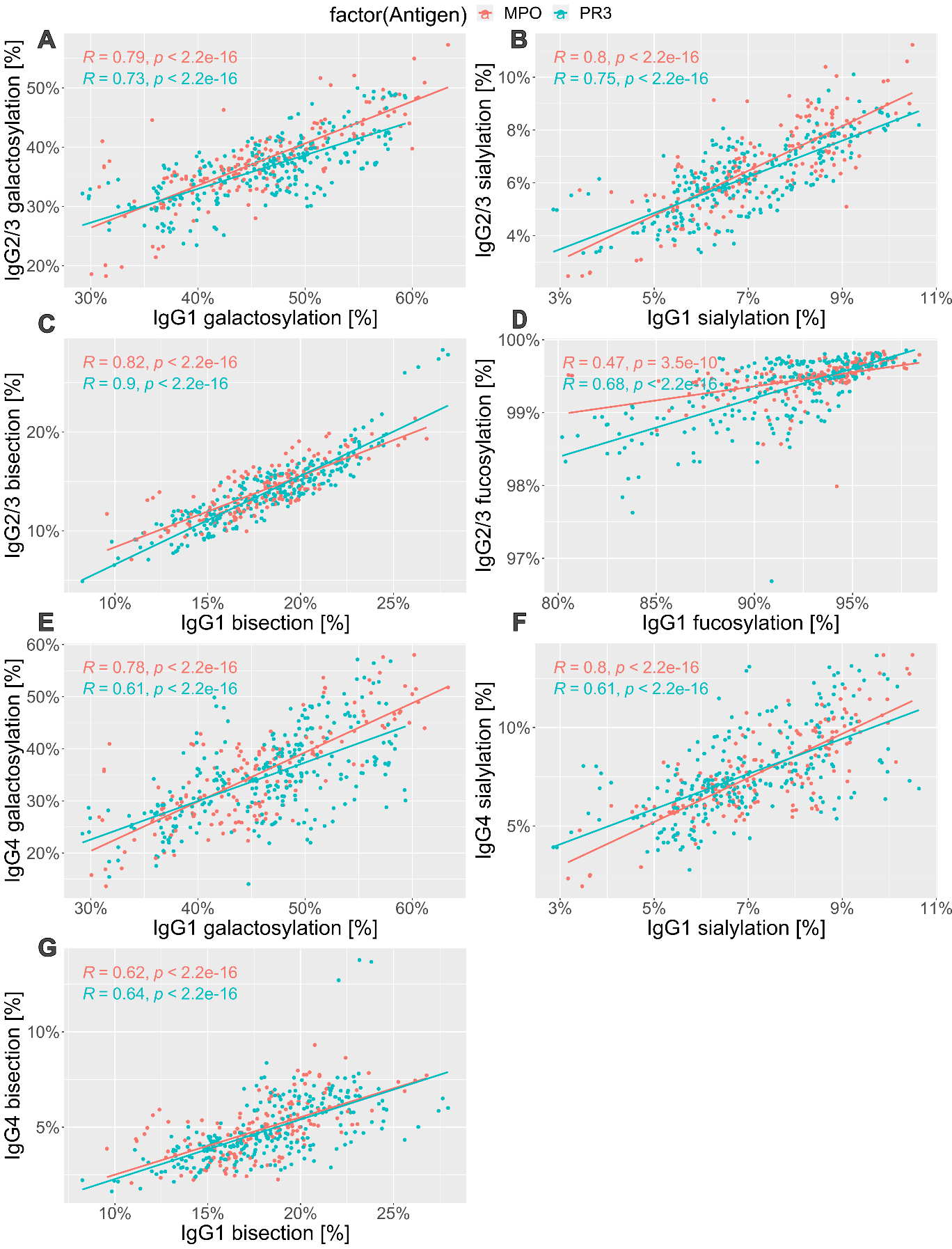


Supplementary Figure 2. Correlation analysis between IgG1 and IgG2/3 Fc (A-D), as well as IgG1 and IgG4 (E-G) Fc glycosylation traits. Data are shown for the regression analysis of the galactosylation, sialylation, bisection, and fucosylation (only for IgG1 and IgG2/3). Spearman’s correlation coefficient and p values are shown for MPO- and PR3-ANCA separately.


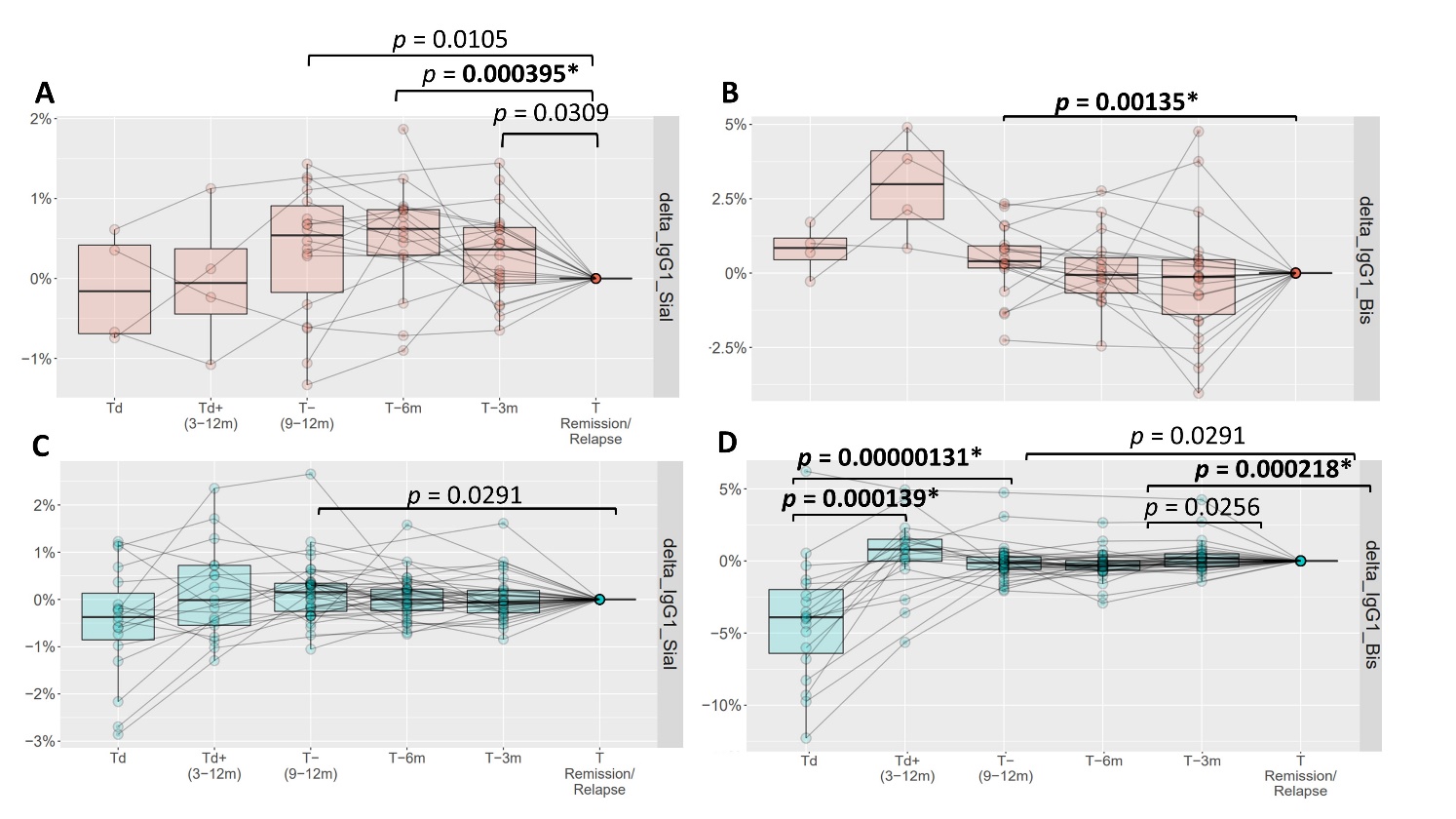


Supplementary Figure 3. Longitudinal changes in ΔIgG1-Fc sialylation (A, C) and ΔIgG1-Fc bisection (B, D) in PR3-postive patients. Differences compared to the point of relapse or time-matched remission are displayed as Δ values, separately for patients who either relapsed (upper panel in red) or stayed in remission (lower panel in green). Significant differences (*p* < 0.0035) are marked with asterisk *. For relapsing patients note: Td and Td+(3-12m) could not be analyzed due to a lack of power.


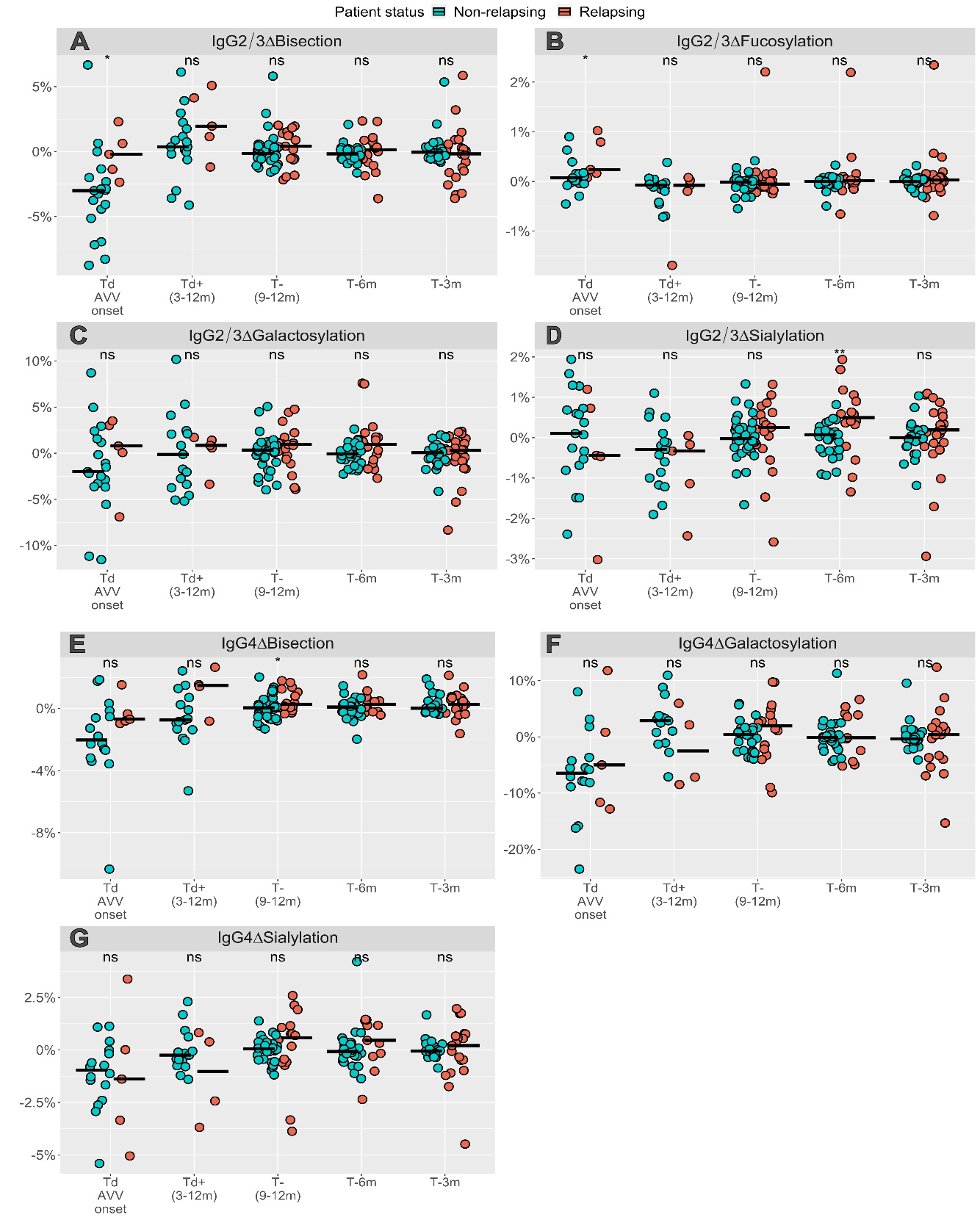


Supplementary Figure 4. Cross-sectional differences in ΔIgG2/3 (A-D) and ΔIgG4 (E-F) Fc glycosylation traits: bisection, fucosylation (only for IgG2/3), galactosylation, and sialylation for PR3-ANCA patients. Differences compared to the point of relapse or time-matched remission are displayed as Δvalues, separately for patients who either relapsed (orange) or stayed in remission (green). Time points range from disease diagnosis (Td AVV onset), and 3-12 months after diagnosis (Td+(3-12m)), 9-12 months (T-(9-12m)), 6 months (T-6m), and 3 months (T-3m) before relapse or time-matched during remission. Median values are indicated by black, horizontal bars. Significant differences are indicated by two asterisks (p < 0.01) or one asterisk (p < 0.05); ns, not significant.

*
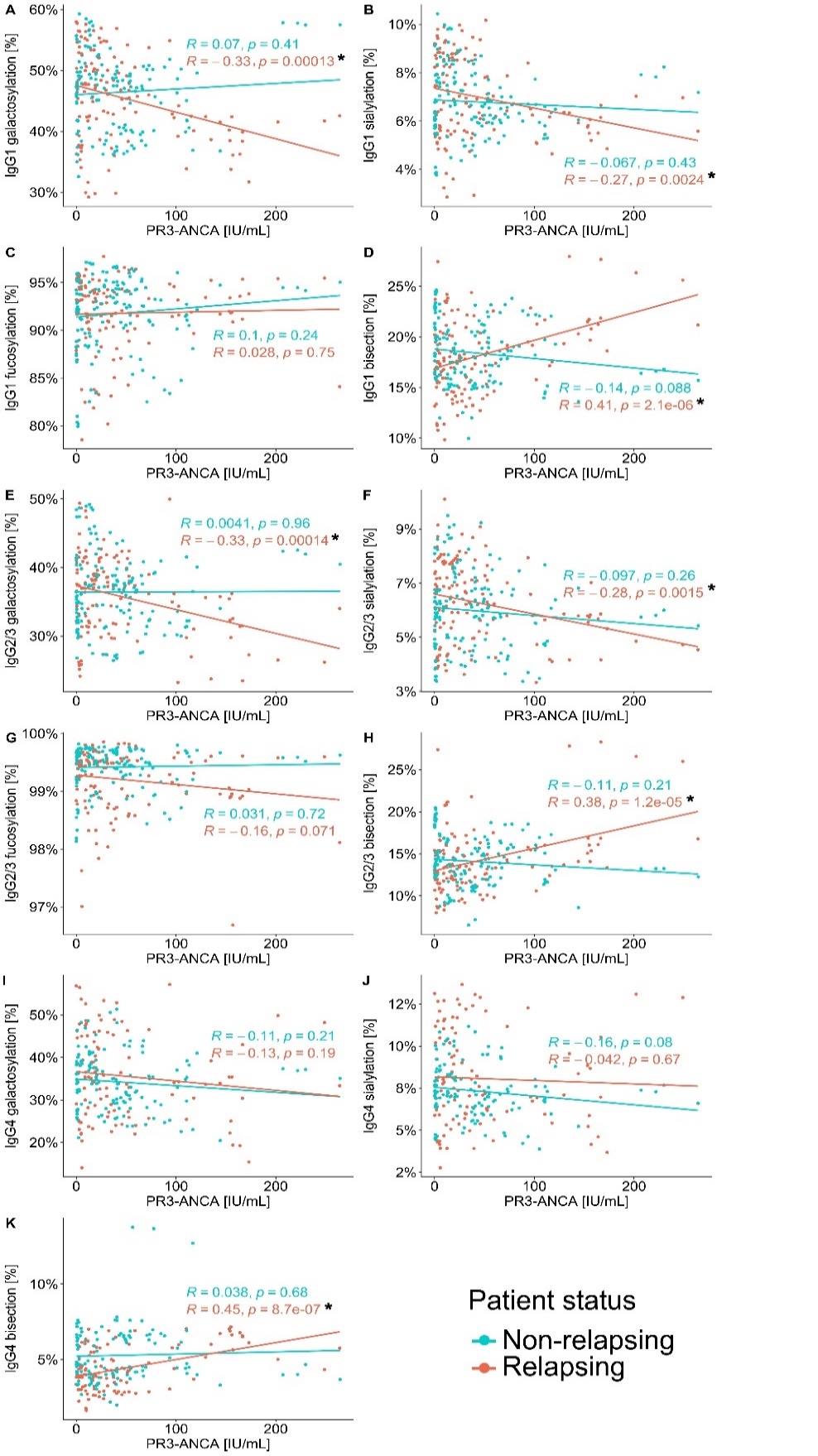
*

Supplementary Figure 5. Correlation analysis between the level of PR3-ANCA titer and IgG glycosylation. Regression analysis was done for IgG Fc-galactosylation, sialylation, fucosylation, and bisection of IgG1 (A-D), IgG2/3 (D-H), IgG4 (I-K), respectively, versus PR3-ANCA titer. Spearman’s correlation coefficient and p values are shown for non-relapsing and relapsing PR3-postive patients separately. Significant differences are indicated by one asterisk (p < 0.05).


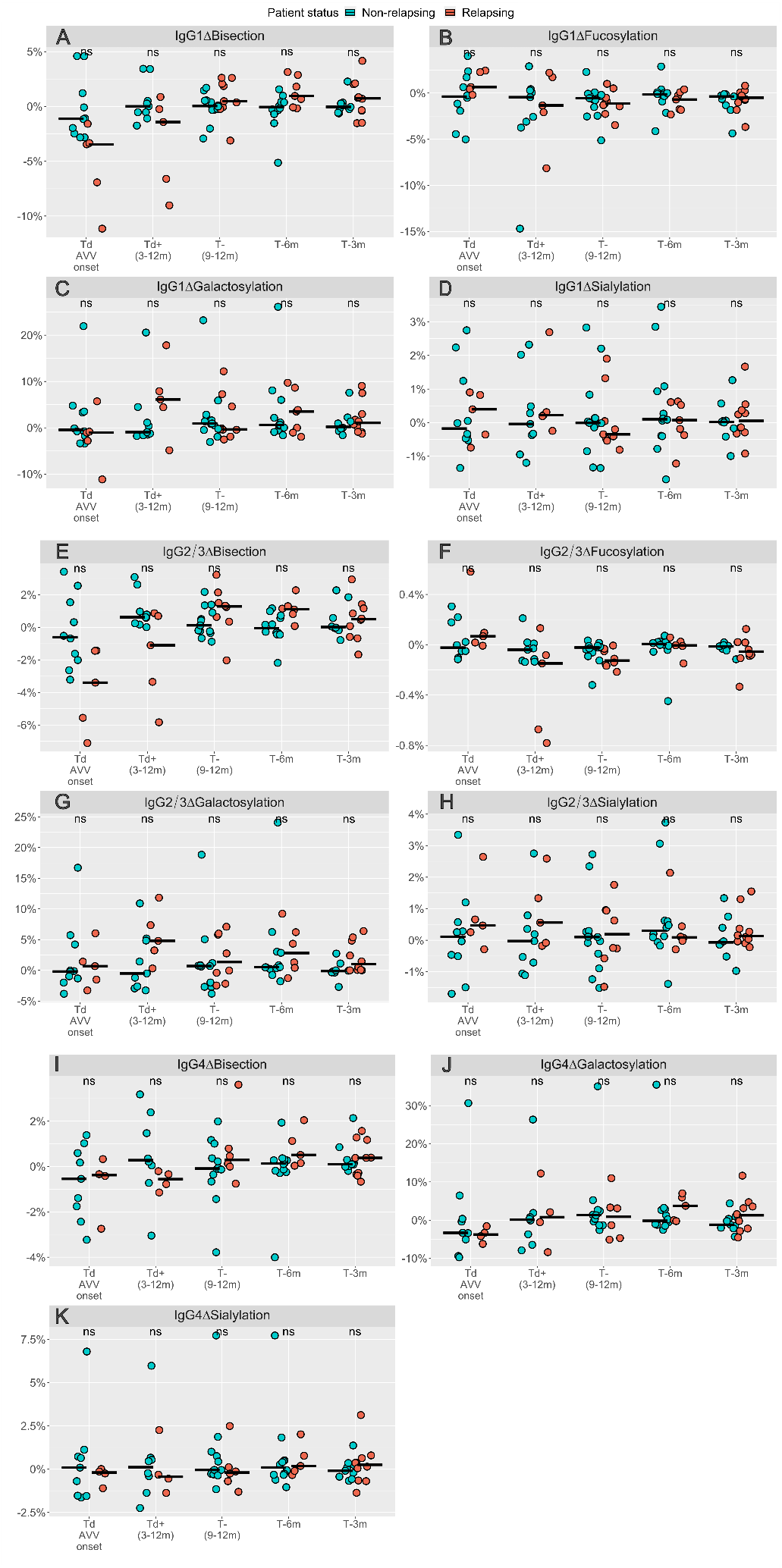


Supplementary Figure 6. Cross-sectional differences in ΔIgG1 (A-D), ΔIgG2/3 (E-F), ΔIgG4 (I-K) Fc glycosylation traits (bisection, fucosylation, galactosylation, and sialylation) for MPO-ANCA patients. Differences compared to the point of relapse or time-matched remission are displayed as Δ values, separately for patients who either relapsed (orange) or stayed in remission (green). Time points range from disease diagnosis (Td AVV onset), and 3-12 months after diagnosis (Td+(3-12m)), 9-12 months (T-(9-12m)), 6 months (T-6m), and 3 months (T-3m) before relapse or time-matched during remission. Median values are indicated by black, horizontal bars. ns, not significant. For Δbisection of IgG1 and IgG2/3 the same difference at Td AVV onset was observed as a trend (Table S6) which was found in GPA-PR3-ANCA patients (Figure 2).


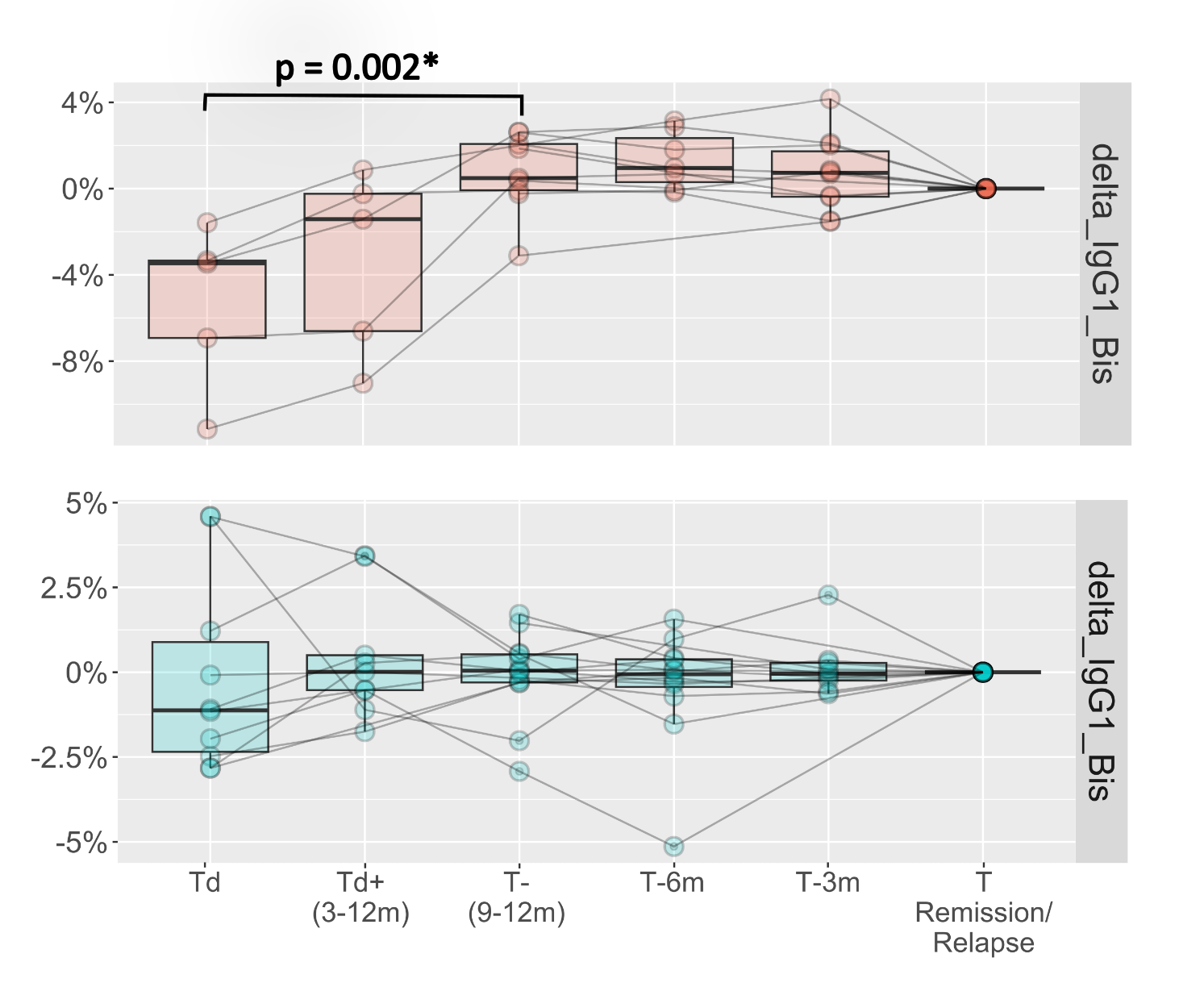


Supplementary Figure 7. Longitudinal changes in ΔIgG1-Fc bisection in MPO-positive patients. Differences compared to the point of relapse or time-matched remission are displayed as Δ values, separately for patients who either relapsed (upper panel in red) or stayed in remission (lower panel in green). Significant differences (*p* < 0.0035) are marked with asterisk *. For relapsing patients note: Td and Td+(3-12m) could not be analyzed due to a lack of power.


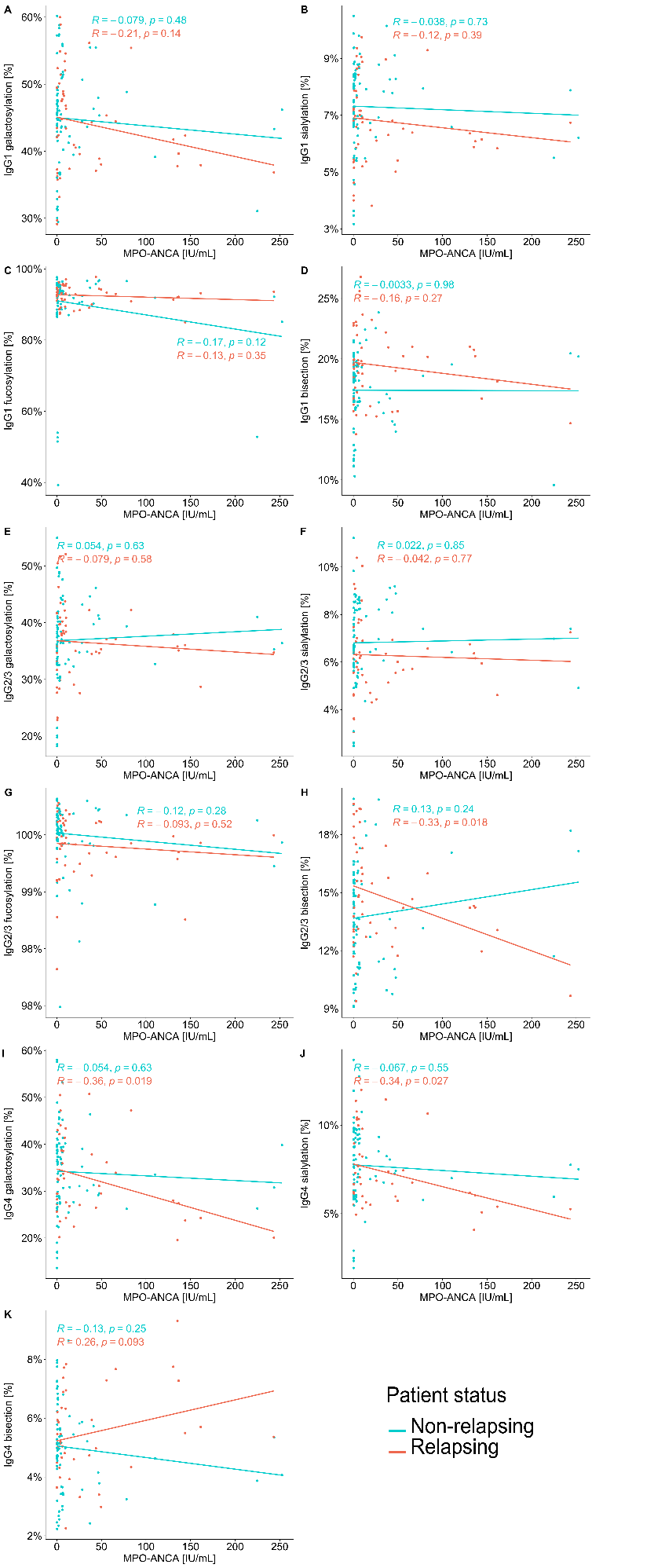


Supplementary Figure 8. Correlation analysis between the level of MPO-ANCA titer and IgG glycosylation. Regression analysis was done for IgG Fc-galactosylation, sialylation, fucosylation, and bisection of IgG1 (A-D), IgG2/3 (D-H), IgG4 (I-K), respectively, versus MPO-ANCA titer. Spearman’s correlation coefficient and p values are shown for non-relapsing and relapsing MPO-positive patients separately.


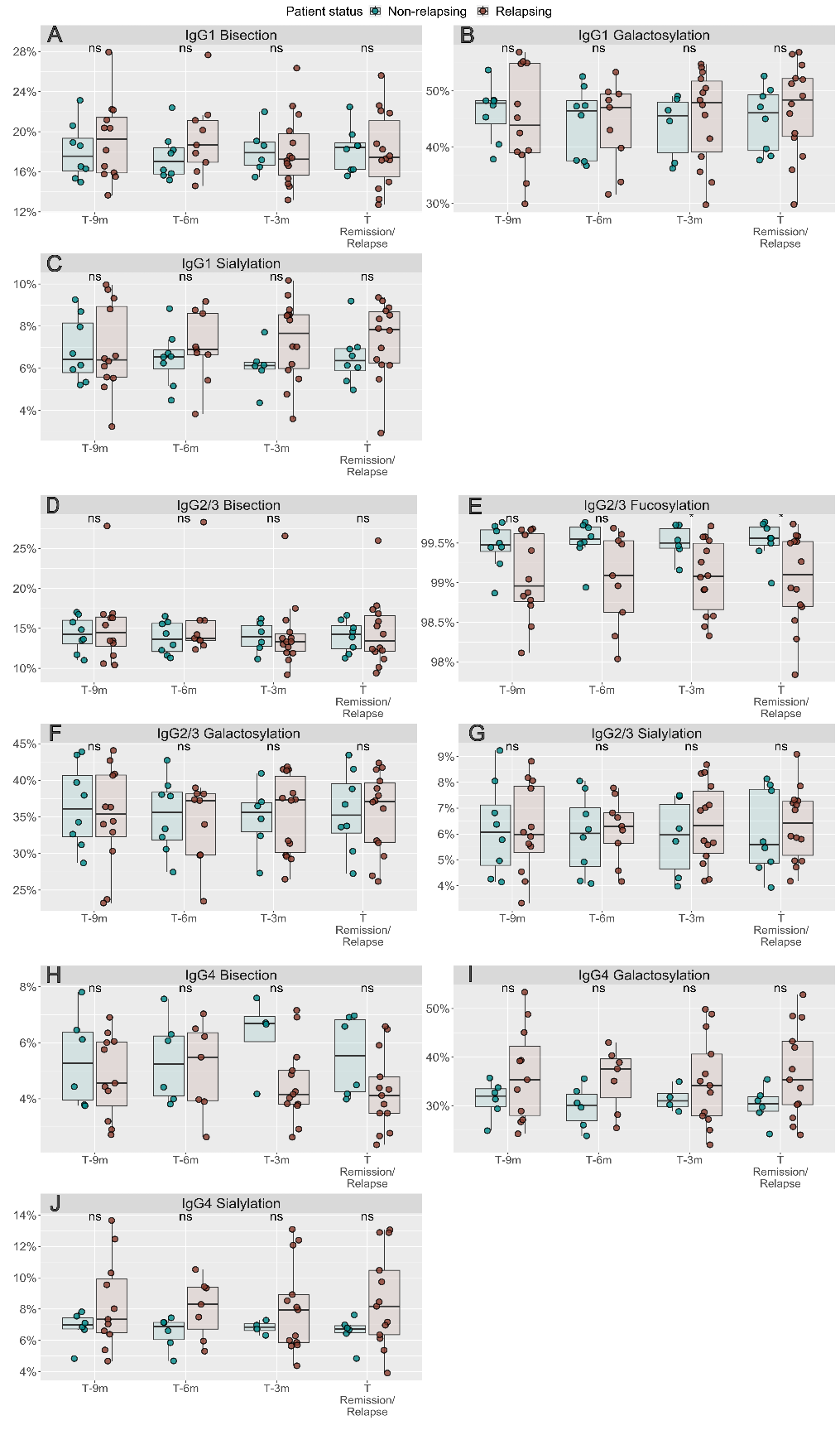


Supplementary Figure 9. Cross-sectional differences in IgG1 (A-C), IgG2/3 (D-G) and IgG4 (H-J) Fc glycosylation traits bisection, galactosylation,sialylation, and fucosylation (only for IgG2/3) for PR3-ANCA patients with an ANCA rise. Total serum IgG1 galactosylation of PR3-ANCA patients who either relapse (brown) and stayed in remission (green), was compared at 9 months, 6 months before, 3 month before and at the time of relapse or remission. Significant differences are indicated by an asterisk (p < 0.05); ns, not significant.


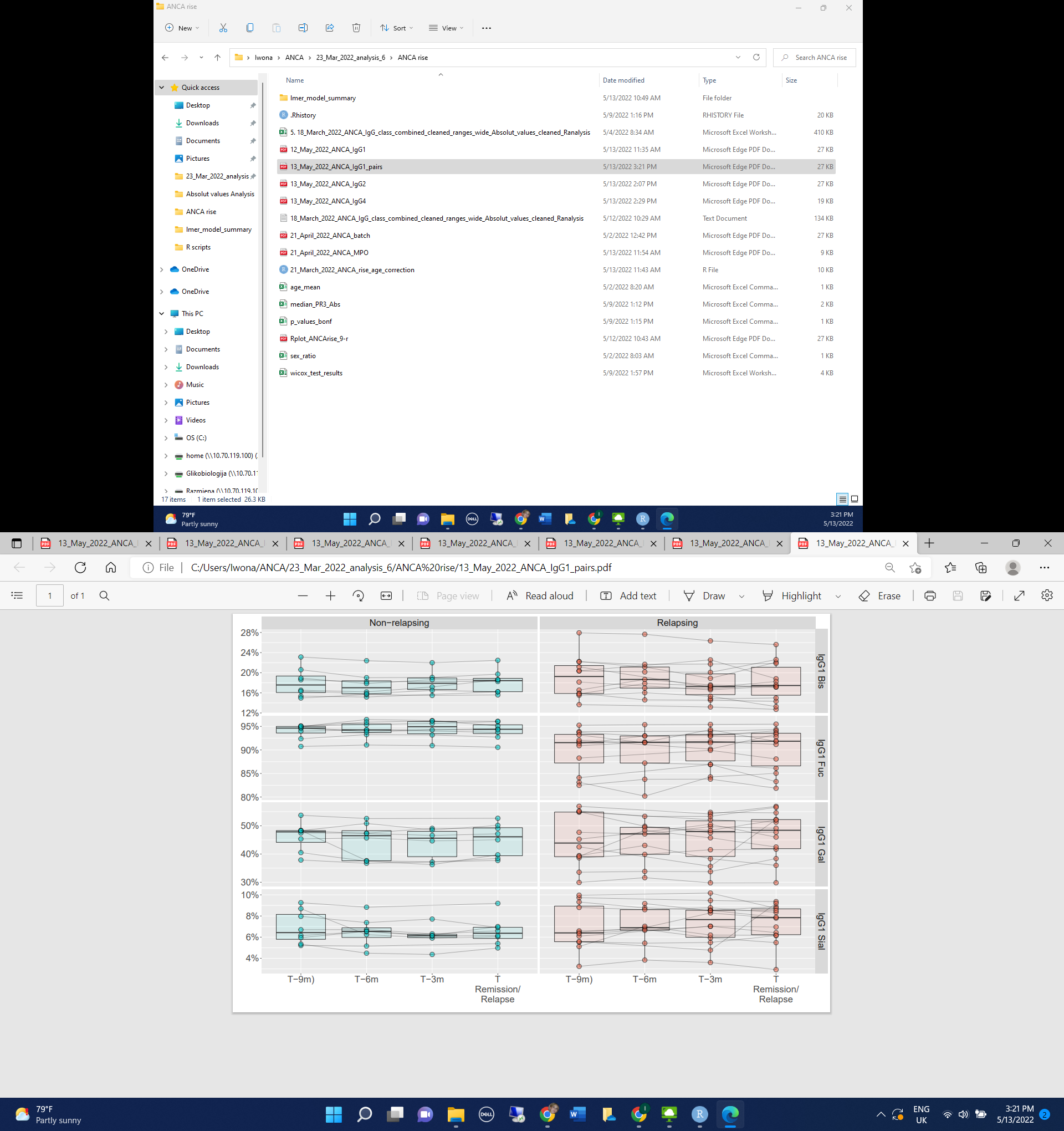


**Supplementary Figure 10.** Longitudinal analysis of IgG1-Fc glycosylation changes for PR3-ANCA relapsing and non-relapsing patients, who experienced a rise in the ANCA titre. IgG1-Fc glycosylation changes are shown for the time points preceding relapse or time-matched remission. Significant differences were evaluated by means of Restricted Maximum Likelihood (REML) with post-hoc Tukey test. There were no significant differences. IgG1 Bis – Bisection of IgG1; IgG1 Fuc – Fucosylation of IgG1; IgG1 Gal – Galactosylation of IgG1; IgG1 Sial – Sialylation of IgG1.


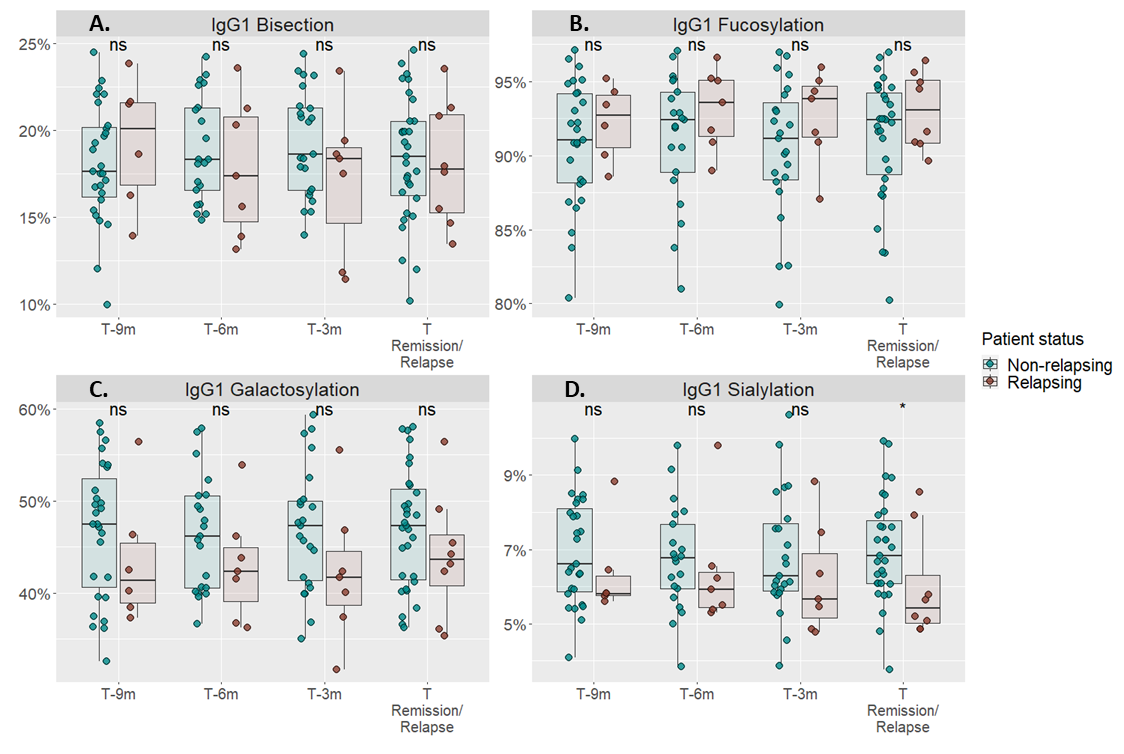


Supplementary Figure 11. Cross-sectional differences in IgG1-Fc bisection (**A**), fucosylation (**B**), galactosylation (**C**), and sialylation (**D**) for PR3-ANCA patients without an ANCA rise. Total serum IgG1 glycosylation of PR3-ANCA patients who either relapse (brown) and stayed in remission (green), was compared at 9 months, 6 months before, 3 month before and at the time of relapse or remission. Significant differences are indicated by an asterisk (p < 0.05); ns, not significant.
